# Inherited human CARD9 deficiency impairs lymphoid cell, but not fibroblast, IL-17–mediated immunity

**DOI:** 10.1172/jci.insight.190875

**Published:** 2026-04-22

**Authors:** Erika Della Mina, Carlos G. El-Haddad, Timothy A. West, Clara W.T. Chung, Jing Jing Li, Vivienne Lea, Elissa K. Deenick, Filomeen Haerynck, Jean-Laurent Casanova, Anne Puel, Cindy S. Ma, Stuart G. Tangye, Alisa Kane

**Affiliations:** 1 Garvan Institute of Medical Research, Darlinghurst, New South Wales, Australia.; 2 School of Clinical Medicine, Faculty of Medicine and Health, UNSW Sydney, Sydney, Australia.; 3 Clinical Immunogenomics Research Consortium Australasia, Darlinghurst, New South Wales, Australia.; 4 Rheumatology Department, Liverpool Hospital, Liverpool, New South Wales, Australia, and University of New South Wales (UNSW) Sydney, Australia, Western Sydney University, Sydney, Australia.; 5 School of Medicine, Western Sydney University, Sydney, New South Wales, Australia.; 6 South Western Sydney Clinical School, Faculty of Medicine and Health, UNSW Sydney, Sydney, New South Wales, Australia.; 7 Immunology and HIV Department, Liverpool Hospital, Sydney, New South Wales, Australia.; 8 Department of Clinical Genetics, Liverpool Hospital, Liverpool, New South Wales, Australia.; 9 School of Women’s and Children’s Health, UNSW, Sydney, New South Wales, Australia.; 10 Department of Anatomical Pathology, Liverpool Hospital, Liverpool, New South Wales, Australia.; 11 Kirby Institute, Faculty of Medicine and Health, UNSW Sydney, Sydney, Australia.; 12 Department of Pediatric Pulmonology, Infectious Diseases and Immunology, and; 13 Primary Immunodeficiency Research Lab, Centre for Primary Immunodeficiency Ghent, Jeffrey Modell Diagnosis and Research Centre, Ghent University Hospital, Ghent, Belgium.; 14 Laboratory of Human Genetics of Infectious Diseases, Necker Branch, INSERM UMR1163, Paris, France.; 15 Paris Cité University, Imagine Institute, Paris, France.; 16 St. Giles Laboratory of Human Genetics of Infectious Diseases, Rockefeller Branch, The Rockefeller University, New York, New York, USA.; 17 Howard Hughes Medical Institute, New York, New York, USA.; 18 Department of Pediatrics, Necker Hospital for Sick Children, Paris, France.; 19 Immunology and HIV Department, St Vincent’s Hospital, Sydney, New South Wales, Australia.; 20 St Vincent’s Clinical School, Faculty of Medicine and Health, UNSW Sydney, Sydney, New South Wales, Australia.

**Keywords:** Genetics, Immunology, Infectious disease, Adaptor proteins, Molecular genetics, T cells

## Abstract

Nearly 100 individuals have been identified who carry deleterious biallelic germline variants in *CARD9* and experience life-threatening, invasive fungal infections caused by *Ascomycetes* but are otherwise resistant to other infectious agents. CARD9 is an adaptor protein expressed predominantly in myeloid cells, which functions downstream of dectin receptors, pattern recognition receptors for fungal antigens, to activate innate immune responses. The impact of CARD9 deficiency on lymphocytes, however, is less clear. We deciphered the functional consequences and delineated mechanisms of disease in a patient (P1) with a nonsense germline homozygous *CARD9* variant (c.673A>T/p.K225*) and invasive *Candida* disease. P1’s PBMCs expressed truncated CARD9 and showed significantly reduced cytokine production in response to fungal ligands. P1 had reduced frequencies of circulating memory CD4^+^ T_H_17-like (CCR6^+^CXCR3^–^) cells. In addition, in vitro differentiation of P1’s naive CD4^+^ T cells into IL-17A/IL-17F–secreting cells was greatly impaired. Consistent with impaired responses of innate and adaptive immune cells from P1 in vitro, proportions of *Candida*-specific CD4^+^ T cells were strongly and selectively diminished. Our findings suggest that the *CARD9* variant identified in P1 is pathogenic, affecting not only CARD9-induced immunity mediated by myeloid cells but also CD4^+^ T cell–intrinsic IL-17–dependent immunity and *Candida*-specific T cell responses.

## Abstract 2

We report a patient suffering from severe fungal infections carrying a novel homozygous nonsense *CARD9* variant (c.673A>T/p.K225*) leading to the expression of a truncated protein unable to mediate innate immunity, CD4^+^ T cell intrinsic IL-17 immunity, and *Candida*-specific T-cell response.

## Introduction

Caspase recruitment domain-containing protein 9 (CARD9) is a signal adaptor molecule expressed predominantly in myeloid cells, including macrophages, DCs, and neutrophils, and at lower levels in other cell types including T cells, B cells (1, 2), hematopoietic (spleen), and nonhematopoietic (brain, lung, liver, heart) tissues (3). CARD9 consists of 536 amino acids and comprises an amino (N)-terminal CARD domain and carboxy (C)-terminal coiled-coil domain (CDD) that mediates protein oligomerization. These domains are shared with other members of the CARD family, CARD10, CARD11, CARD14, which are structurally and functionally homologous to CARD9 (3).

The main function of CARD9 and other CARD-containing proteins is to mediate signal transduction downstream of innate pattern recognition receptors, including C-type lectin receptors (CLRs), intracellular NOD receptors, and nucleic acid sensors, which bind pathogen-associated molecular patterns expressed by microorganisms, thereby ensuring activation of innate immune cells to induce rapid responses against infectious pathogens (4–6). In the setting of fungal infection, the CLRs dectin-1, dectin-2, and Mincle recognize common fungal components (e.g., β-glucan or α-mannose) and utilize CARD9 to initiate an intracellular signaling cascade. This process requires formation of the CBM complex (composed of CARD9, B cell lymphoma 10 [BCL10], and mucosal-associated lymphoid tissue lymphoma translocation protein 1 [MALT1]), followed by downstream activation of the NF-κB pathway. Phosphorylated CARD9 can also interact with Ras proteins (RASGFRF1 and H-RAS) to activate ERK signaling. Both pathways ultimately activate innate immune responses, evidenced by production of proinflammatory cytokines, chemokines, and adhesion molecules (3, 4, 7–14).

Since the first description of autosomal recessive CARD9 deficiency by Gloker et al. in 2009 (15), nearly 100 patients with biallelic germline *CARD9* mutations (OMIM #212050) have been described (1, 2, 15–29). Affected individuals present with susceptibility to life-threatening invasive fungal infections but have no increased risk of bacterial, viral, or parasitic infections (1, 2, 15–28). Interestingly, although there is no single fungal disease common to all patients, each patient is susceptible to infection by *Ascomycetes* species that leads to different clinical outcomes with a broad range of clinical severity. The main clinical manifestations range from superficial chronic mucocutaneous candidiasis (CMC) to fatal invasive *Candida* diseases, severe superficial and deep dermatophytosis, and severe subcutaneous and invasive phaeohyphomycosis (1, 15, 17, 24). A robust and reliable biomarker that predicts disease severity in these patients remains to be identified.

More than 30 germline variants, located in the CARD, CCD, and C-terminal domains of human CARD9 protein have been reported to be disease-causing when inherited as homozygous or compound heterozygous. Patients with CARD9 deficiency show a broad range in age for disease onset (3 to 60 years old), even among those with the same fungal disease, suggesting the clinical penetrance of CARD9 deficiency is not complete until adulthood (1, 2, 16, 18). Expression of mutant CARD9 is variable among different genotypes, ranging from production of full-length protein to an absence of detectable protein (1, 2, 15–28). The immunological phenotype of these patients includes normal numbers of T, B, and NK cells, polymorphonuclear leukocytes, and monocytes. However, unexpectedly, 65% of patients who were tested exhibited eosinophilia and elevated levels of serum IgE (500–20,000 IU/mL) (1, 2, 16, 17). The functional impacts of complete CARD9 deficiency include impaired cytokine and chemokine production by PBMCs, macrophages, and DCs in response to fungal ligands (1, 7, 15, 30, 31). In vivo recruitment of neutrophils to sites of fungal infection were impaired in most patients with CARD9 deficiency who were tested, due to defective production of chemotactic signals by myeloid cells. The few recruited CARD9-deficient neutrophils exhibit reduced oxidative burst and impaired formation of neutrophil extracellular traps (6). However, other studies reported intact oxidative burst by CARD9-deficient neutrophils (4, 30, 32–34). Defective Th17 immunity was found in more than 60% of patients tested, though the mechanism involved remains unclear (1). Beyond the impact of germline biallelic *CARD9* variants, monoallelic *CARD9* variants have been reported as modulators of some inflammatory conditions. Genome-wide association studies identified rare monoallelic *CARD9* variants associated with inflammatory bowel diseases including ulcerative colitis and Crohn’s disease (35–39). Some variants, such as *CARD9* p.S12N (rs4077515), were described as proinflammatory and thus predisposing to disease (38, 39), whereas other variants — *CARD9* c.1434+1G>C (rs141992399) and *CARD9* p.E221K (rs200735402) — were associated with reduced disease incidence (37, 39). By identifying a patient with recurrent invasive *Candida* disease and a homozygous *CARD9* variant, we have now been able to provide greater insight into the mechanisms underlying impaired antifungal immune responses in CARD9 deficiency and further define the cellular requirements for robust immunity against fungal infections.

## Results

### Clinical case.

**Figure 1:**
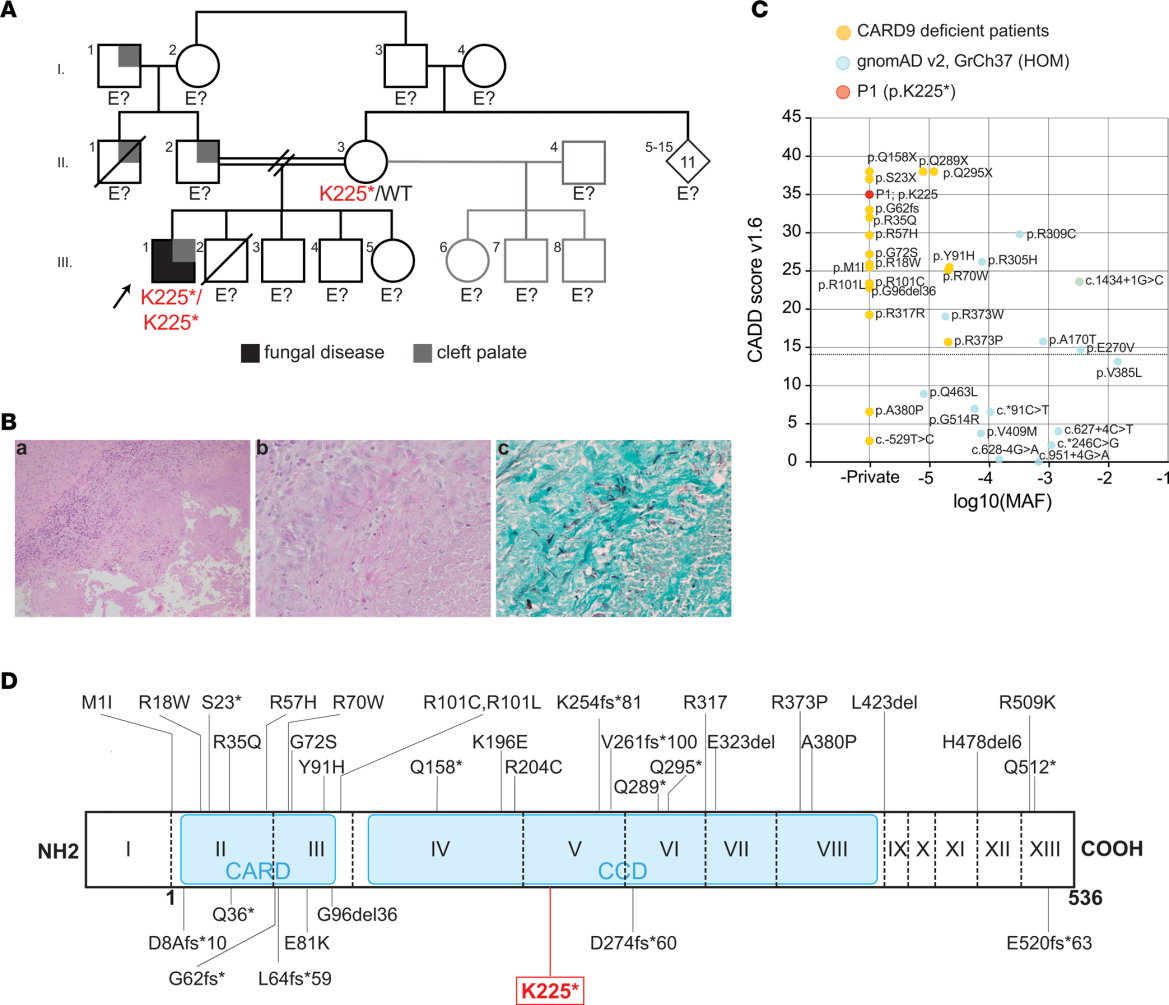
Identification of a homozygous nonsense K225*/K225* variant in CARD9 in a single patient with recurrent fungal infections. (**A**) Pedigree showing familial segregation of the homozygous chr9 (GRCh37):g.139265108T>A, c.673A>T, pK225* substitution in NM_052813.5 exon 5 *CARD9*. Individuals affected by recurrent fungal infections are shown by closed black symbols (III.1, P1), whereas individuals who presented with cleft palate are shown by gray symbols (I.1, II.1, II.2, III.1). (**B**) Histopathological features of the fungus in P1’s biopsy of pretibial lesion: (a) H&E staining showing mixed inflammatory infiltrate including necrotizing granulomas, lymphocytes, plasma cells, neutrophils, and occasional eosinophils; (b, c) fungal organisms with morphology consistent with *Candida* are present in (b) periodic acid Schiff (PAS) and (c) Grocott Methenamine silver stains (original magnification, ×20). (**C**) Minor allele frequency (MAF) and CADD score for variants found in previously reported CARD9-deficient patients (yellow circles), homozygous *CARD9* variants reported in public databases (light blue circles), and variant found in P1 (red circle). The mutation significance cutoff (MSC, 99% CI) is indicated by the dotted line. (**D**) Schematic representation of human CARD9 protein. The main isoform of CARD9 is a 536 aa protein with a CARD domain and a coiled-coil domain (CCD). The proband’s variant (P1) is shown in red; variants identified in previously reported patients with CARD9 deficiency shown in black. The 13 exons are indicated by Roman numerals, and the first exon is nonprotein-coding.

We report the case of a 16-year-old male patient (P1) of Lebanese ancestry, born to consanguineous parents (Figure 1A, III.1, and Table 1), who presented with a 3-month history of progressive left elbow swelling and pain and a 5 cm painful, tender left tibial lesion. No other joints were affected. His background medical history included congenital cleft palate without facial dysmorphism and recurrent episodes of CMC. At 7 years of age, he experienced an episode of sternal candida osteomyelitis. A chest CT scan performed at this time showed a prominent thymus. He did not have a history of other fungal infections, sinopulmonary infections, allergy, or autoimmune disease, nor did he have cardiac disease. He did have an episode of acute disseminated encephalomyelitis in early childhood. However, further details are not known as records of this episode could not be obtained because he had emigrated to Australia at age 13 years with his mother, stepfather, and half-siblings, limiting access to early medical records. Family history was notable for cleft palate affecting his father and paternal grandfather, and a maternal uncle who died of recurrent fevers at 2 years of age. A younger brother died of an undefined neurological condition at age 3 months (Figure 1A). There was no known family history of candidiasis or other immune disease. Investigations of P1 at the time of prerythrocyte sedimentation rate (43 mm/hr) and C-reactive protein (29 mg/L). Total white cell count was 8.8 × 10^9^/L (reference range, 4 × 10^9^ to 10 × 10^9^/L) with normal differential counts including neutrophils (5.2 × 10^9^/L; reference range, 2 × 10^9^ to 7 × 10^9^/L), lymphocytes (2.9 × 10^9^/L; reference range, 1 × 10^9^ to 3 × 10^9^/L) and eosinophils (0.4 × 10^9^/L; reference range, 0.2 × 10^9^ to 1 × 10^9^/L). There were no abnormalities of lymphocyte subsets including CD4^+^ T cell count (0.77 × 10^9/^L; reference range, 0.26 × 10^9^ to 1.32 × 10^9^/L). Levels of IgG (10.8 g/L), IgA (2.34 g/L), and IgM (1.89 g/L) were within the normal range, but IgE was elevated (503 kU/L). P1 had protective levels of IgG against childhood vaccines including tetanus*, Haemophilus influenzae B*, and *Diphtheria*, as well as serum total and neutralizing antibody titers against SARS-CoV-2. Frequencies of SARS-CoV2–binding B cells after vaccination were similar to vaccinated healthy donors. P1 underwent imaging investigations with a CT scan and MRI of the left elbow, which showed effusion and synovitis. Plain radiography of his left tibia and fibula showed a well-defined lucent lesion anterior to the tibia with intact cortex. He underwent surgical debridement of the elbow and tibial lesion. Synovial fluid culture grew *Candida albicans,* and histopathology of the tibial lesion showed fungal elements (Figure 1B). P1 was diagnosed with disseminated candidiasis with candida septic arthritis and commenced a treatment regimen of fluconazole (400 mg daily) for 9 months followed by long-term prophylactic fluconazole (200 mg daily). At the time of last review, 6 years after presentation of septic arthritis at age 16 years, P1 remains well with a single episode of uncomplicated pityriasis versicolor with *Malassezia furfur* at age 18 years, but he has had no further episodes of *Candida* infection or other fungal disease and continues fluconazole prophylaxis.

### Genetics.

Massively parallel sequencing using genomic DNA isolated from the index case P1 (III.1 in Figure 1A) was performed by the Molecular Genetics Department at The Children’s Hospital Westmead (Westmead, New South Wales, Australia) in June 2019. Genes examined belonged to the “Intrinsic and Innate Immunodeficiencies” (v3.0, 92 genes), the “T and B Cell Immunodeficiency” (v3.0, 151 genes), and the “Immune Dysregulation” (v3.0, 56 genes) panels, for a total of 278 unique genes (Supplemental Table 1). This approach identified a homozygous variant in *CARD9* (c.673A>T) and a heterozygous variant in *CHD7* (c.817C>T).

CHD7 (chromodomain helicase DNA-binding 7) is a transcriptional regulator involved in chromatin remodeling, which plays a key role in many developmental pathways (40). Variants in *CHD7* cause a life-threatening disease known as CHARGE (coloboma, heart defects, choanal atresia, growth restriction, genital abnormalities, and ear abnormalities) syndrome (OMIM #214800) (41, 42). More than 1,000 *CHD7* variants have been identified in patients with CHARGE syndrome. The vast majority are de novo and typically lead to absent or reduced CHD7 protein function (43). The *CHD7* heterozygous variant found in P1 (NM_017780.3; c.817C>T) was classified as a variant of unknown significance, and it is absent from P1’s mother (II.3). This variant is reported in a heterozygous state in gnomAD (v4.1.0, https://gnomad.broadinstitute.org/) with an overall allele frequency of 0.000006816 (1/146,713). However, the allele frequency for people of Middle Eastern ancestry, which is relevant as P1 is Lebanese, is 0.0004931 (1/2,027). *CHD7* c.817C>T is also reported in ClinVar (*n* = 1, ID: 1023183) and classified as “likely benign.” Notably, *CHD7* c.817C>T is absent from the CHD7 Database (https://www.chd7.org/), which collects published and unpublished *CHD7* variants and phenotypes and currently contains 554 pathogenic variants from 895 patients (44). Overall, these data suggest that *CHD7* c.817C>T is unlikely to be associated with the infectious phenotype exhibited by P1.

The *CARD9* c.673A>T variant is located in exon 5 (NM_052813.5) (Figure 1D) and changes an AAG codon encoding Lys225 (K225) to a premature stop codon (TAG). The CARD9 p.K225* (hereafter K225*) variant is exclusive to this family, being absent from public databases (gnomADv4.1.0: https://gnomad.broadinstitute.org/, TOPMedBravo: https://bravo.sph.umich.edu). The calculated combined annotation-dependent depletion (CADD) score for *CARD9* c.673A>T is 35, well above the mutation significance cutoff of 14.1, and higher than the CADD score of *CARD9* homozygous variants found in healthy donors in gnomAD (Figure 1C). Furthermore, the CADD/minor allele frequency score of K225* was found to be the fifth highest CADD score among all previously identified disease-causing variants reported in patients with CARD9-deficiency (Figure 1C), thereby establishing this variant as a strong candidate for further investigation. P1’s mother (II.3, Figure 1A) was a heterozygous carrier of the c.673A>T variant (Figure 1A and Supplemental Figure 1A; supplemental material available online with this article; https://doi.org/10.1172/jci.insight.190875DS1). No other relatives were available for genetic testing.

### CARD9 K225* expression and function in HEK293T cells.

**Figure 2:**
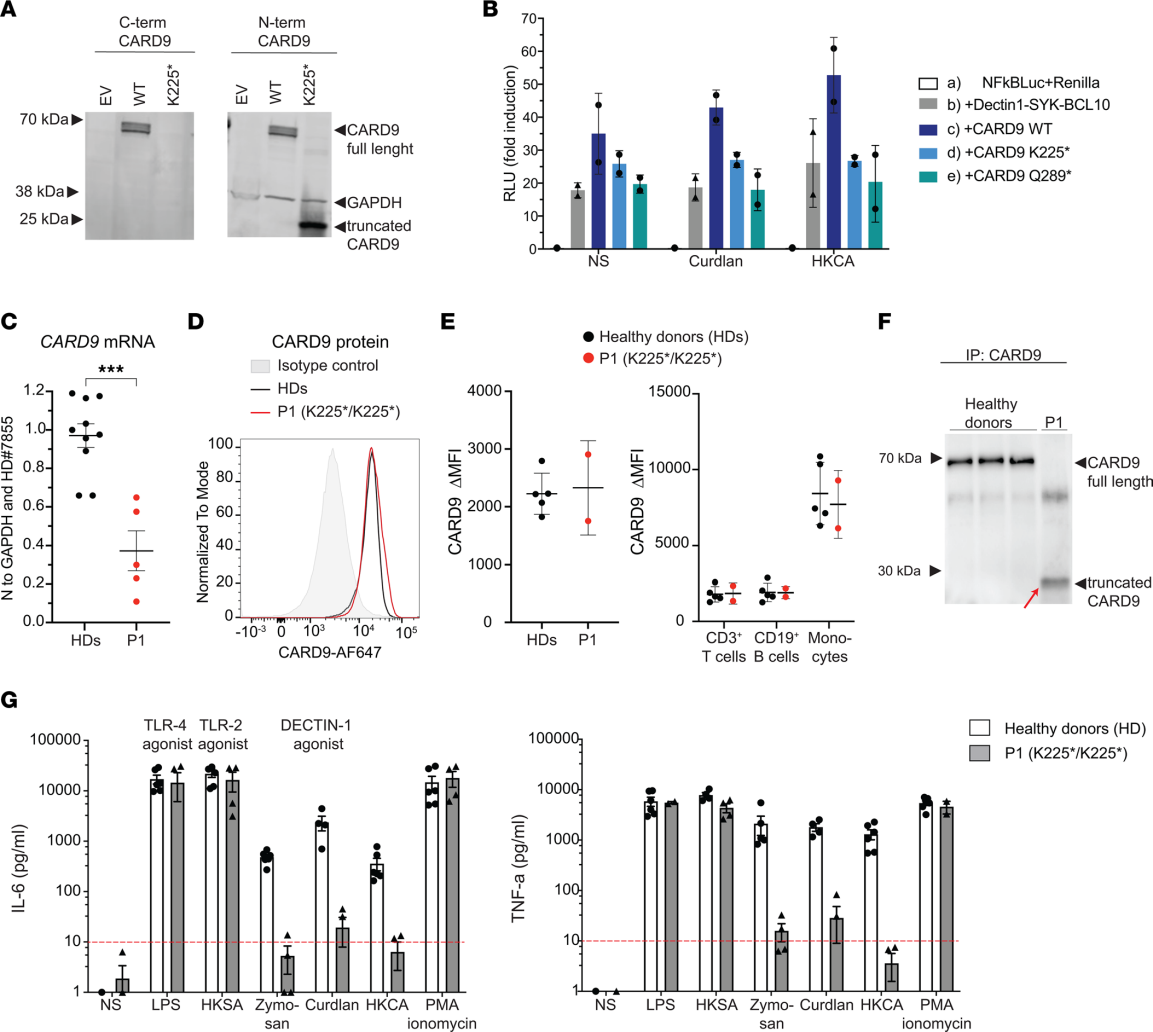
Expression of WT and mutant CARD9 protein in HEK293T cells and patient’s primary cells. (**A**) Total extracts of HEK293T cells transfected with plasmids encoding WT or K225* CARD9 or empty vector (EV) were subjected to Western blotting. CARD9 protein levels were assessed using mAbs specific for CARD9 (left) C-terminal or (right) N-terminal domains and anti-GAPDH (loading control). Similar results obtained in 3 independent experiments. (**B**) NF-κB transcriptional activity in HEK293T cells transfected with reporters alone (a), plus DECTIN1, SYK, and BCL10 (b), plus CARD9 WT (c); or CARD9 K225* (d) or Q289* (e). Cells were stimulated with Curdlan or HKCA. Each symbol represents the ratio of firefly RLU to *Renilla* RLU control. The mean ± SD for 2 independent experiments is shown. NS, not stimulated. (**C**) *CARD9* mRNA levels in PBMCs from HDs (*n* = 10, black) and from P1 (red). Mean ± SD from 4 independent experiments, *P < 0.05 using unpaired 2-tailed *t* test. (**D** and **E**) PBMCs from HDs (black) or P1 (red) were stained with anti-CD3, CD19, CD14, CD16 (surface), and CARD9 (intracellularly) mAbs. CARD9 expression was determined in total lymphocytes (**D** and **E**), T cells, B cells, and CD14^+^CD16^+^ cells (**E**, right panel). The histogram (**D**) depicts CARD9 expression in total lymphocytes from 1 HD (dark gray), P1 (red), and an isotype control (filled light gray). The graphs in **E** represents the mean MFI ± SD of CARD9 (subtracted from MFI of isotype control). (**F**) Western blot showing CARD9 protein levels in total extracts from PBMCs from HDs (*n* = 3) and from P1 after IP. Similar results obtained in 3 independent experiments. (**G**) IL-6 (left) and TNF (right) production by PBMCs after 24 hours of stimulation with the indicated ligands for HDs (*n* = 6, white) and P1(gray). NS, not stimulated. Each data point corresponds to individual HDs or P1 samples, mean ± SEM from 3 independent experiments.

To characterize the impact of the homozygous *CARD9* c.673A>T variant, we studied expression and function of the encoded CARD9 K225* protein. First, we evaluated the consequences of the CARD9 K225* variant on protein expression by Western blot analysis of total cell extracts of HEK293T cells, which lack endogenous CARD9, after transfection with pcDNA3 vectors encoding WT CARD9 protein or the CARD9 K225* variant found in P1. An Ab specific for the C-terminal domain of CARD9 detected a protein at the expected molecular weight of 62kDa in HEK293T cells transfected with WT CARD9 (Figure 2A). In contrast, no signal was detected in protein lysates from cells transfected with CARD9 K225* (Figure 2A). As expected, the same signal in HEK293T cells transfected with WT CARD9 was detected when using an N-terminal anti-CARD9–specific Ab (Figure 2A), which also detected a truncated protein in cells transfected with CARD9 K225* (calculated molecular weight: 25 kDa). This data demonstrated that the *CARD9* c.673A>T homozygous variant encodes a truncated protein (CARD9 K225*) that can be detected in an overexpression system.

Activation of CARD9 in vivo in response to fungal infection ultimately leads to NF-κB activation (3). Thus, we assessed NF-κB transcriptional activity using a reporter assay. HEK293T cells were transfected with (a) NF-κB firefly and *Renilla* reporter vectors (as internal control) alone or in combination with (b) vectors encoding dectin-1, BCL10, and SYK; or vectors from condition (b) plus vectors encoding (c) WT CARD9, (d) CARD9 K225*, or (e) CARD9 Q289*, one of the most frequent mutations reported in patients with CARD9 deficiency (1). Transfected cells were activated for 24 hours with dectin-1 agonists Curdlan and heat-killed *Candida albicans* (HKCA), and then NF-κB firefly and *Renilla* luciferase-specific signals were measured (Figure 2B). NF-κB transcriptional activity in cells transfected to express dectin-1, BCL10, and Syk (Figure 2B, condition [b]) was substantially increased in the presence of WT CARD9 (Figure 2B, condition [c]) in response to both Curdlan and HKCA; it was also higher at basal level (nonstimulated), as previously shown (45, 46). In contrast, CARD9 K225* (Figure 2B, condition [d]) or the previously identified disease-causing variant CARD9 Q289* that served as a control (28, 46) (Figure 2B, condition [e]) failed to amplify NF-κB transcriptional activity, with levels similar to those of cells lacking CARD9 (Figure 2B, condition [b]). These results suggest that in HEK293T cells, despite being expressed at similar levels as full-length protein, truncated CARD9 K225* is unable to induce NF-κB transcriptional activity in response to dectin-1 activation.

### CARD9 expression on patients’ primary cells.

Next, we assessed CARD9 expression in patients’ primary immune cells. First, we measured *CARD9* mRNA in PBMCs isolated from P1 and healthy donors (HDs) (Figure 2C). Using commercial reverse transcription-quantitative PCR (RT-qPCR) primers that span the junction encompassing exons 2 and 3 (upstream of the variant in P1), we found that *CARD9* mRNA was significantly lower in PBMCs from P1 compared with HDs (*n* = 10, Figure 2C). This likely reflects *CARD9* c.673A>T mRNA being subjected to nonsense-mediated decay.

Next, we then determined CARD9 protein expression in PBMCs by flow cytometry using a CARD9 N-terminal domain–specific Ab (Figure 2, D and E). Specificity of this Ab was tested on HEK293T cells transiently transfected with CARD9-expressing vectors (Supplemental Figure 1B). When testing total PBMCs, CARD9 expression was similar in cells from P1 and HDs (average ΔMFI: HDs = 2,220, P1 = 2,330). We then tested CARD9 expression in leukocyte subsets. CARD9 was highly expressed in monocytes and also CD3^+^ T cells and CD19^+^ B cells, albeit at lower levels than monocytes (Figure 2E). This confirms recently published data (2). Importantly, in all subsets tested, CARD9 expression by P1’s cells was comparable to HDs. Lastly, to extend this data, we performed Western blot analysis on PBMC extracts from P1 and HDs (*n* = 3) that had undergone IP with the same N-terminal–specific anti-CARD9 Ab used for HEK293T cells and flow cytometry (Figure 2F). This revealed that immunoprecipitates from HDs contained a protein of approximately 60–70 kDa corresponding to WT CARD9 (Figure 2F). This approximately 60 kDa protein was absent in immunoprecipitates from P1 PBMCs; however, a smaller molecular weight protein (25–30 kDa) corresponding to truncated CARD9 K225* protein was detected (Figure 2F). Together, these results suggest that P1’s PBMCs, despite showing low *CARD9* total mRNA levels, expressed a truncated CARD9 K225* protein at similar levels to full-length WT CARD9 protein detected in HD PBMCs.

### Impact of CARD9 K225* on PBMC response to fungal challenge.

To evaluate the functional consequences of the CARD9 K225* homozygous variant on patient cells, we tested fresh whole blood and PBMCs from HDs (*n* = 6, aged 24–57 years old) and P1 (*n* = 4 independent experiments) for cytokine production after 24 hours of stimulation in the absence or presence of LPS (TLR-4 agonist), heat-killed *Staphylococcus aureus* (HKSA, TLR-2 agonist), Zymosan depleted (an insoluble preparation of *Saccharomyces cerevisiae* cell wall depleted of its TLR-stimulating properties), Curdlan, HKCA, or PMA plus ionomycin (PMA/iono) as a positive control. P1’s PBMCs showed impaired IL-6 and TNF production after stimulation with Zymosan depleted, Curdlan, and HKCA compared with PBMCs from HDs cultured under the same conditions (100-fold difference between P1 and HD) (Figure 2G). In contrast, production of IL-6 and TNF by CARD9^K225*/K225*^ PBMCs in response to LPS (TLR-4), HKSA (TLR-2), or PMA/iono was intact and comparable to HDs (Figure 2G). Similarly, when whole blood from P1 was stimulated with dectin-1 agonists (Zymosan depleted, Curdlan, HKCA), IL-6 and TNF production were strongly impaired (100-fold reduced compared with HD). In contrast, these responses were intact after stimulation with TLR-2 and/or TLR-4 agonists or PMA/iono (Supplemental Figure 1C). Thus, the K225*/K225* variant significantly impairs the ability of CARD9 to activate immune response downstream of dectin-1 in response to fungal-specific antigens.

### Effect of CARD9 K225* in patient’s PBMC immunophenotype.

**Figure 3:**
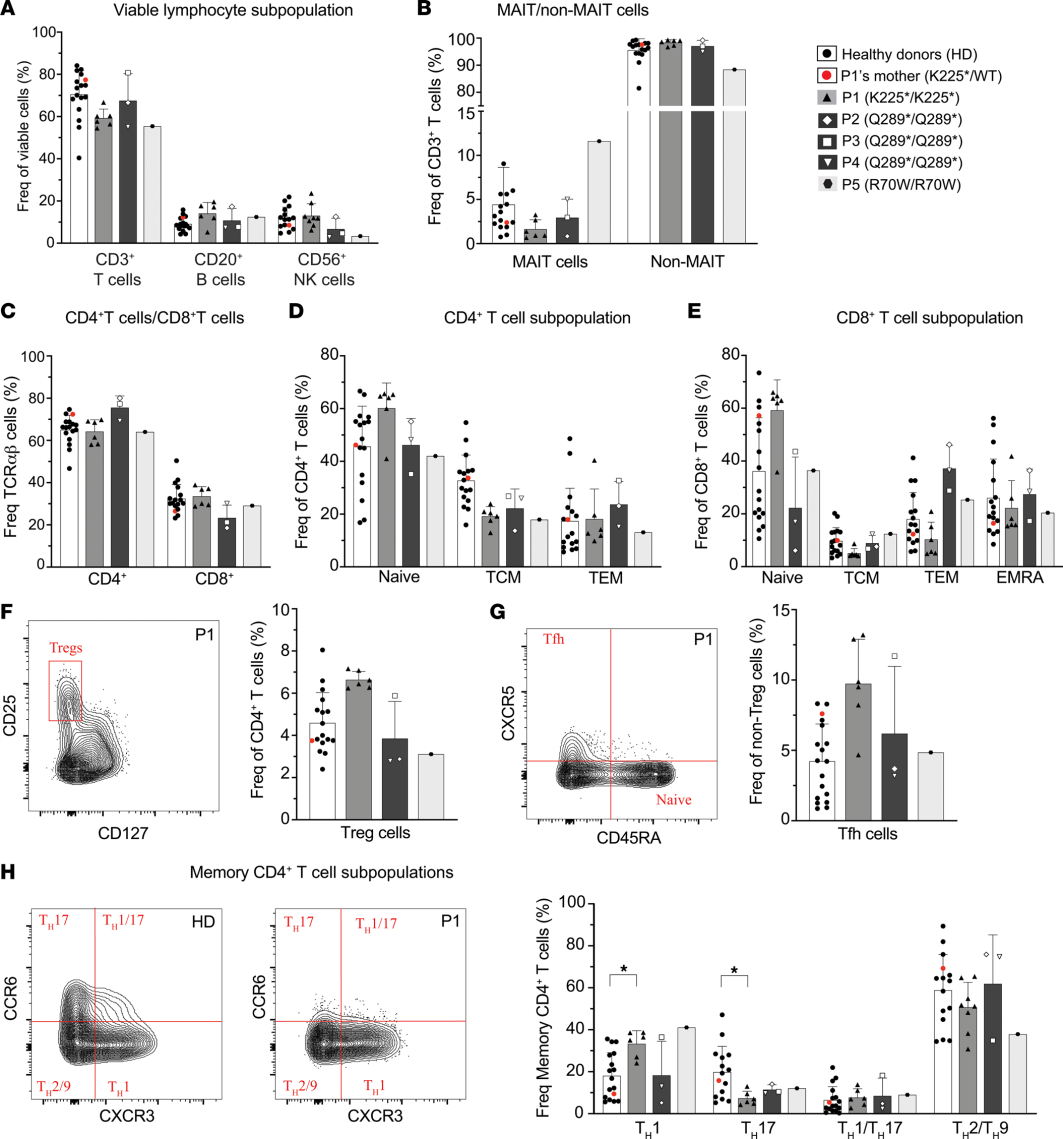
CARD9 K225* affects the frequency of memory CD4^+^ T_H_17 cells. PBMCs from HDs (*n* = 15, white bars), from the heterozygous P1’s mother (CARD9 K225*/WT, red circles among HDs), from the patient with homozygous CARD9 K225* variant (P1, light gray bars), and from previously reported in the literature P2 and P3 (CARD9 Q289*/Q289*, dark gray bars, rhombus and square, respectively) were stained to determine the proportions of (**A**) T cells (CD3^+^), B cells (CD20^+^), and NK cells (CD56^+^); (**B**) MAIT (CD3^+^CD161^+^TCR-Vα7.2^+^) cell frequency; (**C**) CD4^+^ helper (CD4^+^ CD8^–^) and CD8^+^ cytotoxic (CD4^–^CD8^+^) T cell frequency; (**D**) CD4^+^ helper T cell subsets: naive (CD4^+^CCR7^+^CD45RA^+^), central memory (T_CM_, CD4^+^CCR7^+^CD45RA^–^), effector memory (T_EM_, CD4^+^CCR7^–^CD45RA^–^); (**E**) CD8^+^ cytotoxic T cells subsets: naive (CD8^+^ CCR7^+^CD45RA^+^), T_CM_ (CD8^+^ CCR7^+^CD45RA^–^), T_EM_ (CD8^+^ CCR7^–^CD45RA^–^), effector revertant memory (T_EMRA_, CD8^+^ CCR7^–^CD45RA^+^). (**F**) Representative contour plots gated on P1 showing frequency of Tregs (CD25^hi^CD127^lo^) (left panel) and proportion of Tregs (CD25^hi^CD127^lo^) in HDs, P1’s mother, P1–P5 (right panel). (**G**) Representative contour plots gated on P1 showing Tfh frequency (CD25^lo/–^CXCR5^+^CD45RA^–^) (left panel) and corresponding histograms showing Tfh proportion in HDs, P1’s mother, P1–P5 (right panel). (**H**) Representative contour plots gated on 1 HD and P1 showing frequency of T_H_1 (CD25^lo/–^CXCR5^–^CD45RA^–^CXCR3^+^CCR6^–^), T_H_17 (CD25^lo/–^ CXCR5^–^CD45RA^–^CXCR3^–^CCR6^+^), T_H_2/9 (CD25^lo/–^CXCR5^–^CD45RA^–^CXCR3^–^CCR6), T_H_1/17 (CD25^lo/–^CXCR5^–^CD45RA^–^CXCR3^+^CCR6^+^) (left panel) and corresponding histograms showing proportion of T_H_1, T_H_17, T_H_2/9, T_H_1/17 in HDs, P1’s mother, P1, P2, and P3 (right panel). The data represent the mean ± SD of 6 independent experiments. *P < 0.05 using ANOVA or Kruskal-Wallis test.

To identify the impact of CARD9^K225*/K225*^ on the phenotype of PBMCs, we examined immune cell subsets in HDs (*n* = 15), P1 (*n* = 6, 6 samples collected over 5-years and analyzed in 6 independent experiments), P1’s mother (CARD9^K225*/WT^, II.3 Figure 1A), 4 previously reported CARD9-deficient patients: P2 (46), P3 (28), and P4 (47) are homozygous for the CARD9 Q289* nonsense variant; P5 is homozygous for the CARD9 R70W variant (48) (Table 1). P1 had normal proportions of total T cells, B cells, and NK cells (Figure 3A). Moreover, proportions of MAIT, CD4^+^, and CD8^+^ T cells (Figure 3, B and C), as well as CD21^lo^, transitional, naive, and memory B cells (Supplemental Figure 2, A–C) and of total as well as CD56^hi^ and CD56^lo^ NK cells (Supplemental Figure 2D) were all comparable to HD cells. Within the CD4^+^ T cell compartment, P1 showed increased proportions of naive (60.2% vs. 45.6% in HDs; mean 46.2% in P2–P4, 42% in P5), decreased proportions of central memory (T_CM_) cells, and intact proportions of T_EM_ cells (Figure 3D). Similarly, proportions of naive CD8^+^ T cells were increased in P1 and CD8^+^ T_EM_ cells were decreased compared with HDs and CARD9^Q289*/Q289*^-deficient patients P2–P5 (Figure 3E). Frequencies of CD4^+^ Treg and T follicular helper cells in all patients with CARD9 deficiency tested (P1–P5) were similar to HDs (Figure 3, F and G). Interestingly, within the memory CD4^+^ T cell subpopulation, P1 (CARD9^K225*/K225*^) consistently showed significantly decreased proportions of T_H_17-type cells (CCR6^+^CXCR3^–^; 7.4% vs. 20% in HDs, P1’s heterozygous mother, II.3, 15.8%) compared with HDs, a finding similar to that in the additional CARD9-deficient patients tested (P2–P5, range from 9.78% to 14.1%) (Figure 3H). In contrast, P1 showed slightly increased T_H_1 cells (CCR6^–^CXCR3^+^; 32.3% in P1 vs. 18.1% in HDs 18.2% in P2–P4) compared with HDs and patients P2–P4 but a similar level to P5 (41%) (Figure 3H). The heterozygous mother II.3 (CARD9 _K225*/WT_) showed cell frequencies largely comparable to HDs for each cell subset tested (Figure 3, A–H, red circles).

### CARD9 K225* affects production of Th17-type cytokines by memory and naive CD4^+^ T cells in vitro.

**Figure 4:**
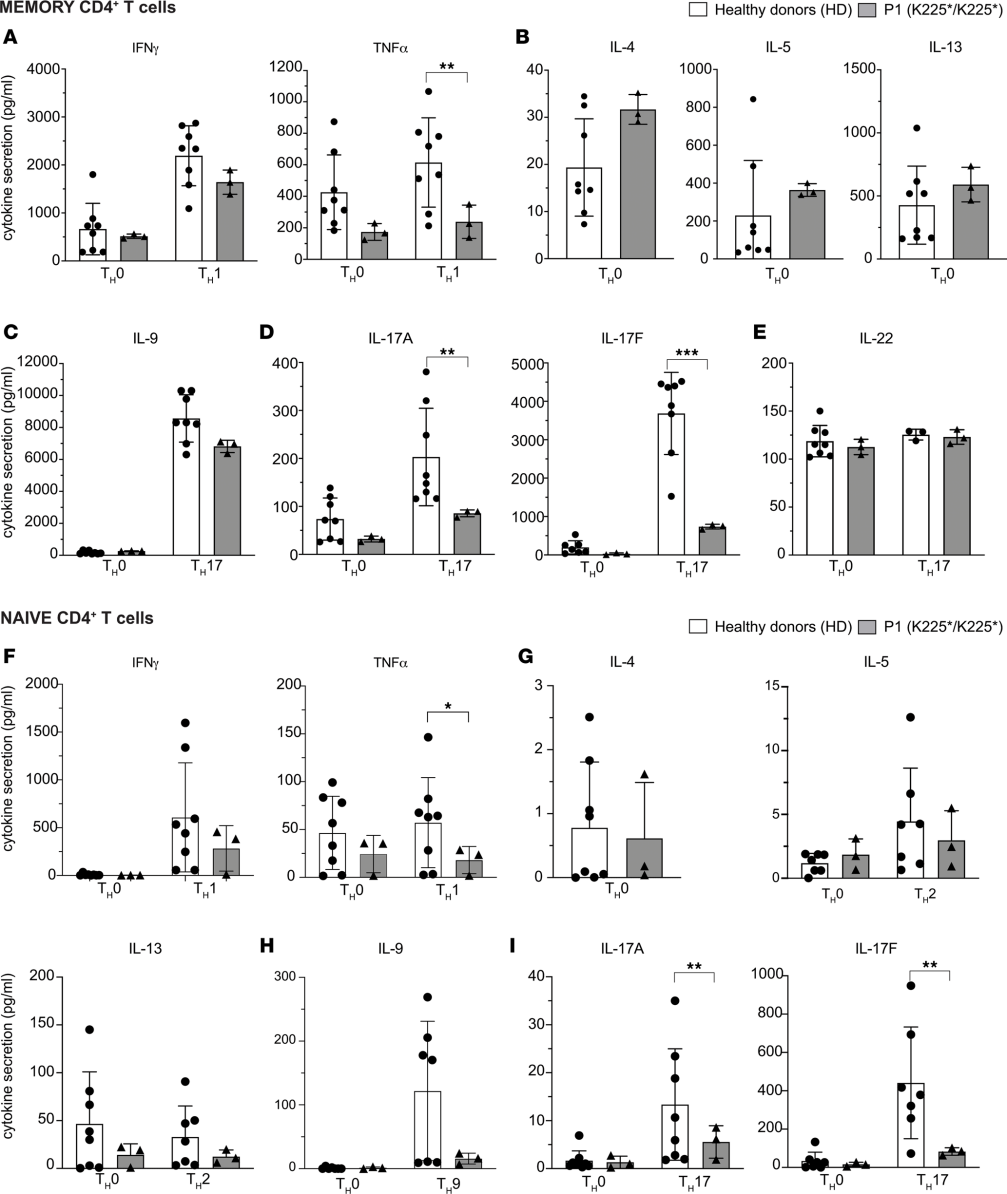
CARD9 K225* memory and naive CD4^+^ T cells have impaired T_H_17 polarization. Sort-purified memory (**A**–**E**) and naive (**F**–**I**) CD4^+^ T cells from HDs (*n* = 8) and P1 (*n* = 3 experiments) were cultured for 5 days with TAE beads under T_H_0, T_H_1, T_H_17-polarizing conditions. (**A**–**E**) Memory CD4^+^ T cell secretion of (**A**) T_H_1, (**B**) T_H_2, (**C**) IL-9, and (**D**) T_H_17 cytokines were determined by cytometric bead arrays. (**E**) IL-22 expression was determined by ELISA. (**F**–**I**) Production of (**F**) T_H_1 cytokines, (**G**) T_H_2 cytokines, (**H**) IL-9, and (**I**) T_H_17 cytokines by naive CD4^+^ T cells from HDs and P1 under T_H_0 and the indicated polarizing culture conditions were determined by cytometric bead arrays. The mean ± SD for 3 independent experiments is shown. *P < 0.05 using a Mann-Whitney *U* test.

Approximately 60% of patients with reported CARD9 deficiency and susceptibility to fungal infection exhibited impaired T cell–dependent IL-17–mediated immunity (1). To decipher the potential impact of the CARD9 K225* homozygous variant on CD4^+^ T cell function, we first sort-purified memory (CD45RA^–^) CD4^+^ T cells from healthy donors (*n* = 8) and P1 (*n* = 3 independent experiments), which were then stimulated under different polarizing conditions for 5 days. After this time, expression and secretion of T_H_1 (IFN-γ, TNF-α), T_H_2 (IL-4, IL-13, IL-5), T_H_9 (IL-9), T_H_17 (IL-17A, IL-17F, IL-22), and T_FH_ (IL-21) cytokines were determined (Figure 4 and Supplemental Figure 3).

Memory CD4^+^ T cells from P1 exhibited preserved production but reduced secretion of IFN-γ and TNF-α under T_H_1 conditions (Figure 4A and Supplemental Figure 3A). Production of IL-2 (not shown), IL-4, IL-5, IL-13, and IL-21 by CARD9^K225*K225*^ memory CD4^+^ T cells was comparable to HDs (Figure 4B and Supplemental Figure 3, B and E), suggesting T_H_2 and T_FH_ polarization is not affected by CARD9 deficiency. Under T_H_17 conditions, IL-9 production from P1 remained at the lower end of the range observed in HDs (Figure 4C and Supplemental Figure 3C). In contrast, P1’s memory CD4^+^ T cells showed significantly decreased production of the canonical T_H_17 cytokines IL-17A and IL-17F under T_H_0 and T_H_17-polarizing conditions (Figure 4D and Supplemental Figure 3D). Interestingly, IL-22 was almost intact (Figure 4E and Supplemental Figure 3D). Thus, CARD9 deficiency results in a selective defect in the ability of memory CD4^+^ T cells to differentiate into T_H_17-type cells in vivo.

To determine whether these Th17 cytokine-specific defects in CARD9^K225*/K225*^ memory CD4^+^ T cells are caused by extrinsic defects such as impaired induction by myeloid cells or are T cell intrinsic, we tested the ability of sort-purified naive CD4^+^ T cells from P1 and HDs to differentiate into T_H_1, T_H_2, T_H_9, and T_H_17-type cells under in vitro culture conditions (Figure 4, F–I, and Supplemental Figure 3, F–H). Similar to ex vivo cytokine production by P1 memory CD4^+^ T cells, we observed decreased but not abolished production of T_H_1 (IFN-γ, TNF-α) and T_H_9 (IL-9) cytokines and intact production of T_H_2 (IL-4, IL-13, IL-5) cytokines compared with HDs (Figure 4, F–H, Supplemental Figure 3, F–H). Furthermore, differentiation of CARD9^K225*/K225*^ naive CD4^+^ T cells into IL-17A– and IL-17F–secreting cells under Th17 polarizing conditions was significantly impaired (Figure 4I). These data suggest that a CD4^+^ T-cell–intrinsic defect contributes to the impaired secretion of Th17 cytokines shown by CARD9^K225*/K225*^ memory CD4^+^ T cells.

### CARD9-deficient CD4^+^ T cells have impaired Candida-specific T cell responses.

**Figure 5:**
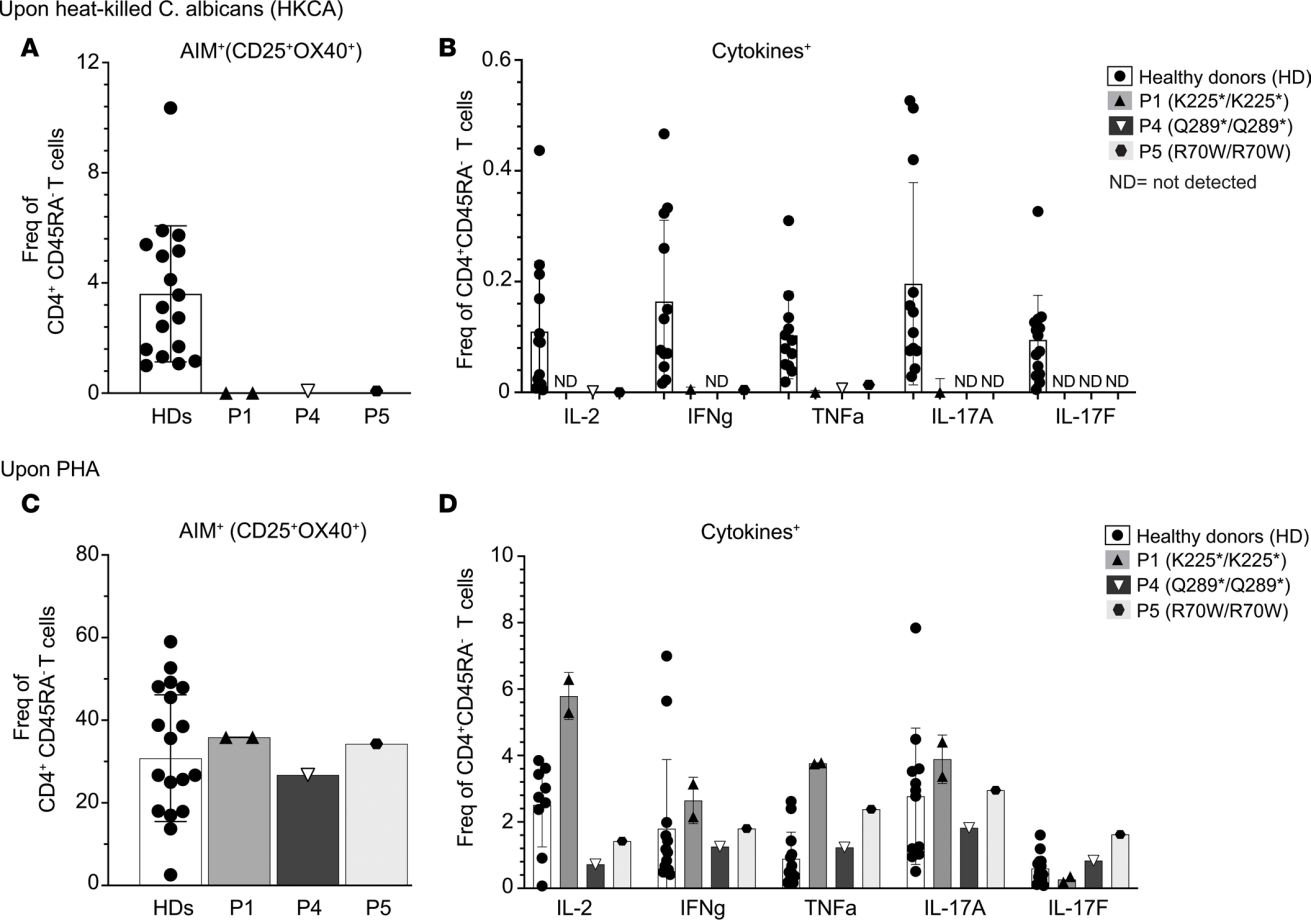
Defect in *Candida*-specific T cell response by P1’s CD4^+^ T cells. HDs (*n* = 20), P1 CARD9^K225*^ (*n* = 2 samples), P4 CARD9^Q289*^ (*n* = 1), and P5 CARD9^R70W^ (*n* = 1) patient PBMCs were stimulated in vitro with heat-killed *C*. *albicans* (HKCA) (**A** and **B**) or PHA (**C** and **D**). Frequency of Ag-specific CD4^+^ T cells measured by flow cytometry are reported. Total viable OX40^+^CD25^+^ CD4^+^ T cells upon HKCA (**A**) or PHA (**C**) were background-subtracted against nonstimulated culture (negative control). Brefeldin A was added during the last 4 hours of stimulation, and intracellular staining of IL-2, IFN-γ, TNF-α, IL-17A, and IL-17F cytokines was performed (**B** and **D**); frequency of cytokine-producing cells upon HKCA (**B**) or PHA (**D**) was background-subtracted against non-stimulated culture. ND, not detected. Each point represents a different individual (for HDs) or sample (P1, P4, and P5); mean ± SD for 3 independent experiments is shown.

To further assess the functionality of CARD9^K225*/K225*^, we performed activation-induced marker assays to quantify *Candida*-specific cells by assessing induction of OX40 and CD25 coexpression on CD4^+^ T cells in PBMCs in response to in vitro challenge with HKCA, as previously described (49, 50). These assays were also performed on PBMCs from P4 (CARD9^Q289*/Q289*^) and P5 (CARD9^R70W/R70W^) (Table 1), 2 previously described patients with CARD9 deficiency, to establish whether the results were unique to P1 or a generalized defect affecting other unrelated cases of CARD9 deficiency. *Candida*-specific CD45RA^–^OX40^+^CD25^+^ T cells were detected in all HDs tested (*n* = 18, range: 1.07%–10.4% of CD4^+^CD45RA^–^, Figure 5A). In stark contrast, frequencies of *Candida*-specific CD45RA^–^CD4^+^ T cells in PBMCs from P1, P4, and P5 were extremely low, similar to the nonstimulated conditions (negative control; Figure 5A). We next assessed intracellular cytokine expression by *Candida*-specific CD4^+^ T cells present in these cultures. Consistent with the previous results, *Candida*-specific CD4^+^ T cells producing IL-2, IFN-γ, TNF-α, IL-17A, and IL-17F were consistently observed in all tested HDs (Figure 5B). In contrast, *Candida*-specific CD4^+^ T cells producing these cytokines were not detectable in PBMCs from P1, nor in PBMCs from P4 or P5 (Figure 5B). To confirm that impaired responses of CARD9-deficient CD4^+^ T cells did not result from a general inability to upregulate OX40 and CD25 or produce cytokines during short-term culture, we also assessed CD4^+^ T cell responses after activation with the polyclonal mitogen phytohemagglutinin (PHA). In response to PHA, similar proportions of CD45RA^–^OX40^+^CD25^+^ (Figure 5C), as well as cytokine-producing T cells, were detected in PBMCs from HDs and P1, P4, and P5 (Figure 5D). These data establish that CARD9 deficiency is associated with an inability to elicit antigen-specific CD4^+^ T cell responses after exposure to *Candida*. Such a defect is independent of the disease-causing variant, as this was observed for all CARD9-deficient patients who were tested (p.K225* from P1, p.Q289* from P4, and p.R70W from P5).

### Human fibroblasts do not respond to fungal challenge in vitro.

**Figure 6:**
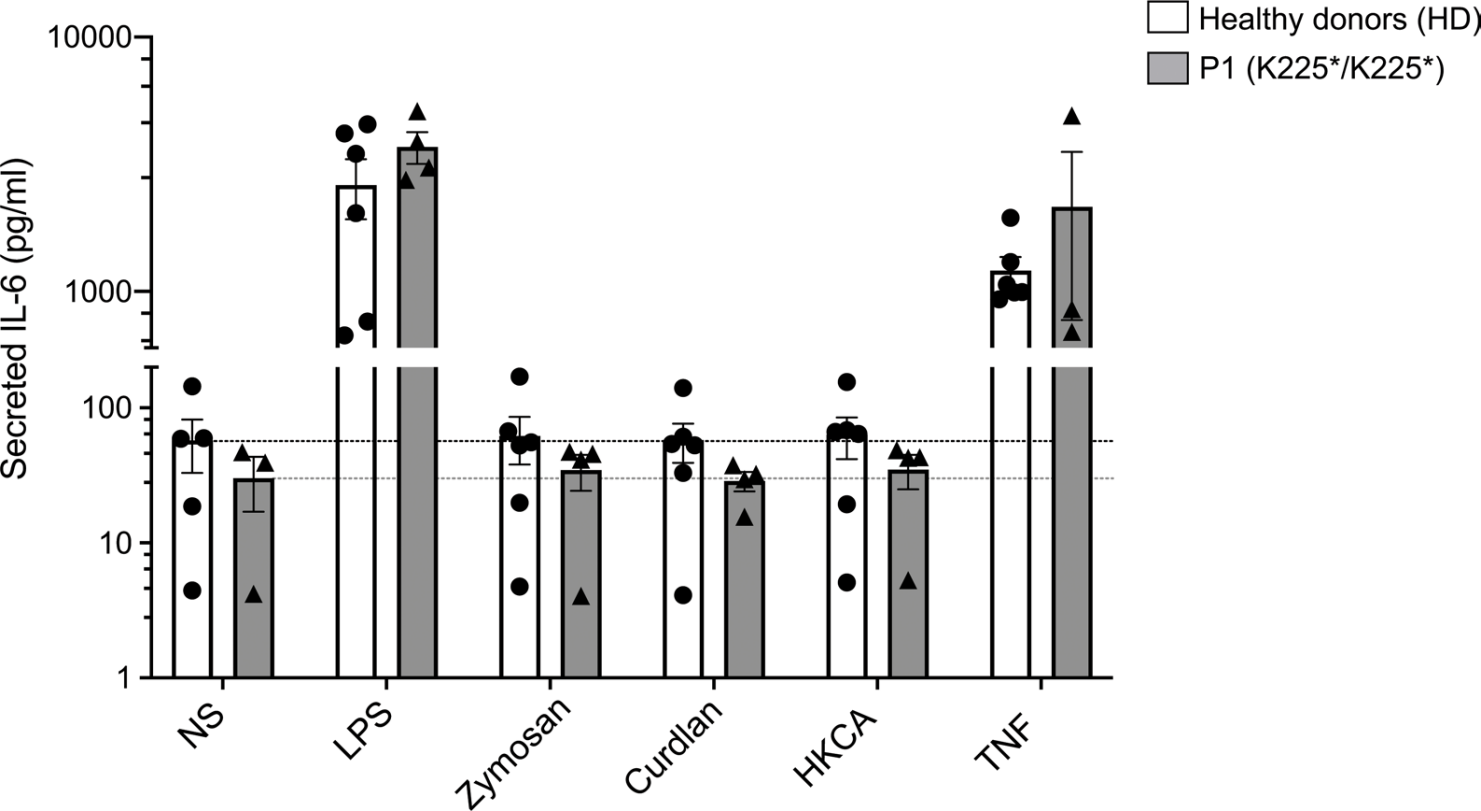
CARD9 K225* primary fibroblast response to fungal-specific antigens. IL-6 production by primary fibroblasts from HDs (*n* = 2) and P1 after 24 hours of stimulation with LPS (TLR-4 agonist), Zymosan depleted, Curdlan, HKCA (all dectin-1 agonists), and TNF. Results shown as mean ± SEM for 3 independent experiments. The horizontal dotted lines indicate IL-6 production in primary fibroblast culture with media only (condition NS, nonstimulated cells).

Both invasive and superficial fungal disease (i.e., CMC, dermatophytosis, phaeohyphomycosis) typical of patients with CARD9 deficiency largely involve the skin (1, 2, 17, 18, 21, 26, 45, 46). Despite this, there is a paucity of compelling data regarding the role of immune responses elicited in the skin in these patients. The skin consists of the epidermis, dermis, cutaneous appendages, and subcutaneous tissue. The epidermis contains keratinocytes, melanocytes, and Langerhans cells; the dermis houses a larger population of specialized immune cells including macrophages, DCs, NKs, mast cells, CD4^+^ T cells, γδ T cells, and nonhematopoietic cells such as fibroblasts. A previous study showed that in human primary fibroblasts, NF-κB activation downstream of dectin-1 (as well as TLR2/6 and TLR4) was BCL-10 dependent (51). Other studies have suggested that in keratinocytes, the CBM complex downstream of dectin-1 consists of BCL10, MALT1, and CARD14, while in other nonhematopoietic cells, CARD10 may be part of the CBM complex (52). To clarify the requirement for CARD9 in responses of skin cells, we generated primary fibroblasts from P1 and HDs (*n* = 2). Primary fibroblasts were cultured for 24 hours in the absence or presence of LPS (TLR-4 agonist), Zymosan depleted, Curdlan, HKCA (dectin-1 agonists), and TNF as a positive control (Figure 6). CARD9-deficient fibroblasts from P1 produced IL-6 to the same level as CARD9-sufficient fibroblasts from HDs after stimulation with either LPS or TNF (Figure 6). In contrast, stimulation of primary fibroblasts from either HDs or P1 with any of the dectin-1 agonists tested (depleted Zymosan, Curdlan, or HKCA) had no effect on IL-6 production over that observed for unstimulated fibroblasts. These results suggest that impaired host defense in patients with CARD9 deficiency is predominantly due to defective immunity in leukocytes rather than nonhematopoietic cells, though characterization of other skin cells would further refine this conclusion.

## Discussion

The role of CARD9 in innate immunity against fungal pathogens has been intensely studied in mice and humans (1). There are now approximately 100 reported patients with CARD9 deficiency (15, 19–27, 45, 46), the vast majority of whom have experienced life-threatening invasive fungal infections, whereas others present with superficial fungal disease (CMC and dermatophytosis). These phenotypes are caused by *Candida* spp, dermatophytes, or black fungi. Notably, the immune impairment in patients with CARD9 deficiency is fungal-specific and they do not have increased susceptibility to most if any other microorganisms (1, 2, 17, 18, 21, 26). The study of patients with CARD9 deficiency has suggested CARD9 is important in myeloid cells for activating NF-κB and MAPK signaling downstream of CLRs specific for fungal recognition (1). Impaired cytokine and chemokine production in response to stimulation with various fungal ligands was shown for CARD9-deficient whole blood cells, PBMCs, monocytes, monocyte-derived macrophages, and monocyte-derived DCs (1, 2, 17, 18, 21, 26, 45, 46). Although it has been reported that T cell–dependent IL-17–mediated immunity is impaired in most patients with CARD9 deficiency who were tested (1, 30), these previous results failed to provide clear evidence of specific cellular mechanisms responsible for aberrant antifungal immunity because assessment of IL-17 production or responses are typically measured using total PBMCs — rather than defined cell subsets — from patients with CARD9 deficiency. Thus, in contrast to the established defects in myeloid cells, much less is known about the consequences of biallelic pathogenic *CARD9* variants on human lymphocytes, as well as precise mechanisms of susceptibility to invasive fungal infections in CARD9 deficiency.

Here, we investigated a homozygous premature stop codon variant in *CARD9* (c.673A>T; p.K225*) and determined its impact on the phenotype and function of hematopoietic and nonhematopoietic cells. The *CARD9* c.673A>T variant led to expression of a truncated protein (CARD9^K225*/K225*^) both in transfected HEK293T cells and patients’ PBMCs. The pathogenicity of this variant was confirmed using patient PBMCs, which produced 100-fold less IL-6 and TNF in response to stimulation with fungal ligands than PBMCs from healthy donors. We also observed a reduced frequency of circulating T_H_17 cells in P1, consistent with the general conclusion that blood T_H_17 cells are reduced in at least 50% of patients with CARD9 deficiency (1). However, despite these findings, the mechanism(s) by which a lack of functional CARD9 affects T_H_17 cell differentiation in humans remains unclear. Importantly, data derived from assessing responses of sorted naive and memory CD4^+^ T cell subsets in vitro provide some insights. In fact, a major finding from our study is not only an impairment of IL-17A/IL-17F cytokine production by memory CARD9^K225*/K225*^ CD4^+^T cells under neutral (T_H_0) and T_H_17-inducing culture conditions (Figure 3), but, critically, naive CARD9^K225*/K225*^ CD4^+^ T cells poorly differentiate into IL-17A– and IL-17F–secreting cells in vitro under T_H_17 polarizing conditions. Thus, our data strongly indicate that the T_H_17 defects result from an intrinsic defect in CD4^+^ T cells, and this would be compounded by T cell–extrinsic defects in the form of a lack of availability of T_H_17-promoting cytokines produced by myeloid cells (Figure 2G). Combined, these cooperative defects manifest as a paucity of T_H_17-cytokine–producing *Candida-*specific CD4^+^ T cells in patients with CARD9 deficiency. Additional studies are required to elucidate how a defect of CARD9 could translate to an intrinsic defect of CD4^+^ T cell differentiation in vivo.

The optimal management of fungal disease in CARD9 deficiency is unknown (1, 53). Patients are typically treated with an initial therapeutic course of antifungal agents followed by life-long prophylaxis treatment. Surgical interventions might be required for extensive or deep dermatophytosis and fungal abscesses. In a limited number of patients with CARD9 deficiency (*n* = 5), GM-CSF has been trialed with variable outcomes (54, 55), thus rendering it difficult to draw firm conclusions about the efficacy of this treatment to rescue impaired CARD9-dependent immunity. Allogeneic-hematopoietic stem cell transplantation (HSCT) is a curative treatment option for severe inborn errors of immunity where the defect originates in hematopoietic stem cells. Because the mortality rate of patients with CARD9 deficiency is significant (13.3%) (1) and recurrent invasive fungal infections lead to high morbidity in affected patients, alternative therapies for the most severe and treatment-refractory cases are required. The role of HSCT as a treatment option in CARD9 is currently unclear. To date, there are 3 published cases of patients with CARD9 deficiency with very severe disease and poor quality of life (intraabdominal aspergillosis, deep/invasive dermatophytosis, all unresponsive to multiple antifungal therapies) (34, 53), who have undergone HSCT, albeit with mixed outcomes: 2 patients successfully achieved clinical remission but 1 died due to HSCT-related complications. A concern in undertaking such therapeutic decisions in patients with CARD9 deficiency is the lack of data regarding the expression and role of CARD9 in tissue-resident immune cells, epithelial cells, and other nonhematopoietic cells. Our study assesses the requirement for CARD9 in regulating cytokine production by primary fibroblasts. IL-6 induction in response to TLR4 and TNF-R agonists was similar for fibroblasts isolated from HDs or the CARD9^K225*/K225*^ patient. Interestingly, stimulation with dectin-1 agonists did not result in detectable increases in cytokine production by WT or CARD9^K225*^ fibroblasts. This suggests fibroblasts might have a limited role in mediating antifungal immunity in the absence of other dermal cells and may explain successful outcomes after HSCT in 2 of the 3 CARD9-deficient patients. Additional experiments are required to understand how these in vitro results translate into in vivo protective immunity against fungal infections and to further improve our understanding of the role of other skin cells in CARD9-mediated immunity. This knowledge will be of relevance in considering HSCT as a curative treatment option for patients with CARD9 deficiency.

In summary, we discovered that the *CARD9* (c.673A>T) variant encodes a truncated protein in P1’s PBMCs. Although CARD9 K225* is expressed at similar levels as WT full-length CARD9 protein in HDs, it is unable to activate signaling downstream of dectin-1, thus failing to mediate immune responses against fungal infections. Moreover, we found decreased frequencies of circulating memory CD4^+^ CCR6^+^ T_H_17 cells in P1’s PBMCs and impaired in vitro differentiation of P1’s naive CD4^+^ T cells into IL17-secreting cells under T_H_17 polarizing conditions. Furthermore, *Candida*-specific CD4^+^ memory T cells were undetectable in the peripheral blood of P1 and other patients with CARD9 deficiency. Combined, our results reveal intrinsic and extrinsic functions of CARD9 in human CD4^+^ T cell differentiation, thereby providing substantial insight into mechanisms underlying impaired antifungal immunity in autosomal recessive CARD9 deficiency.

## Methods

### Sex as a biological variable.

In this study, the index patient (P1) carrying the *CARD9* variant was male. Control samples were obtained from age-matched HDs of both sexes, and no significant differences related to sex were observed amongst the HDs. Sex was not considered as a biological variable in the statistical analysis due to the single-patient nature of the study.

### Genetics.

Genomic DNA was obtained from whole blood from patients. The CARD9 variant was amplified from genomic DNA by PCR (primer sequence available upon request). Amplicons were purified by centrifugation through Sephadex G-50 Superfine resin (Amersham-Pharmacia-Biotech) and sequenced with the BigDye Terminator Cycle Sequencing kit (Applied Biosystems), and sequences were analyzed with an ABI Prism 3500 (Applied Biosystems). The sequences obtained were aligned with the genomic sequence of CARD9 (Ensembl) with SnapGene 8.0 software.

### Primary peripheral blood samples.

PBMCs were isolated by Ficoll-Hypaque centrifugation (Merck) from cytapheresis obtained from patient P1, his relatives, or HDs. Cells were either used fresh or cryopreserved and stored in liquid nitrogen until use. We analyzed P1’s samples collected at 7 time points when the patient was between 18 and 24 years of age. HDs were aged 28 to 54 years old depending on the experiment (Figure 2, C–E, and G: 28 to 54 years old, Figure 2F: ages 28, 31, 54; Figure 3, Figure 4, and Figure 5: 28–40 years old). Although the age range of the HDs was greater than the age of P1 at the times of testing, it has been established that proportions and absolute numbers of peripheral blood lymphocytes reach the adult level starting from approximately 16 years of age (56). Primary fibroblasts were obtained from punch skin biopsy specimens and cultured in DMEM (Gibco) supplemented with 10% FBS (Gibco).

### Overexpression of CARD9 variants.

The HEK293T cell line was purchased from American Type Culture Collection (ATCC) and cultured in DMEM supplemented with 10% FBS (Gibco). HEK293T cells were grown at 37°C, under an atmosphere containing 5% CO_2_. The plasmid containing pcDNA3 CARD9 cDNA (NM_052813.5) was provided in-house. Constructs carrying single-nucleotide mutant alleles were generated from these plasmids by mutagenesis with appropriate primers, with the Q5 Site-Directed Mutagenesis kit (E0552S, New England Biolabs), according to the manufacturer’s protocol. Plasmids were amplified in competent *E*. *coli* cells (One Shot TOP10 Chemically Competent, C404003, Thermo Fisher Scientific). HEK293T cells were plated in 6-well plates at a density of 500,000 cells per well and incubated overnight. The next day, cells were transiently transfected with the various constructs in the presence of Opti-MEM (Thermo Fisher Scientific) and Lipofectamine 3000 transfection reagent (L3000-015, Thermo Fisher Scientific) according to the manufacturers’ instructions.

### Whole blood and PBMC activation.

Blood samples were collected in tubes containing heparin and tested, generally after 24 hours of transport at room temperature. Blood was diluted 1:2 in RPMI 1640 medium (GIBCO BRL, Invitrogen). Aliquots of diluted blood were dispensed into a 48-well plate and incubated at 37°C, under an atmosphere containing 5% CO_2_, under different sets of conditions — with medium alone (nonstimulated), with agonists of dectin-1: zymosan depleted (InvivoGen tlrl-zyd; zymosan depleted is obtained by treating zymosan, an insoluble preparation of *Saccharomyces cerevisiae* cell wall, with hot alkali to remove its TLR-stimulating properties). Therefore, zymosan depleted (5 μg/mL) activates the C-type lectin receptor dectin-1 but not TLR2, HKCA (InvivoGen, tlrl-hkca, 10^6^ particles/mL), Curdlan (InvivoGen, tlrl-curd, 50 μg/mL), TLR-4 agonist LPS *Salmonella enterica* serotype Minnesota Re 595 (Sigma-Aldrich, 10 ng/mL), TLR-2 agonist HKSA (InvivoGen, tlrl-hksa, 10^7^ particles/mL), or with PMA/iono (0.2 μg/mL and 2 × 10^–4^ μg/mL, respectively). Supernatants were collected after 24–48 hours and stored at –20°C until their use for cytokine (IL-6 and TNF) determination by cytometric bead array (BD Biosciences).

### NF-κB luciferase assay.

NF-κB luciferase activity was assessed as previously described (18, 45, 46). Briefly, we transiently transfected HEK293T cells with 100 ng of NF-κ B–dependent firefly luciferase vector, 40 ng of *Renilla* luciferase vector (pRL-SV40-d238) as an internal control, 10 ng of dectin-1 vector, 10 ng of Syk vector, 10 ng of BCL10 vector, and 10 ng of either CARD9 WT or CARD9 K225* or CARD9 Q289* vector (110). Transfection was performed using the Lipofectamine LTX kit (Life Technologies,15338100), according to the manufacturer’s instructions. HEK293T cells were then stimulated for 24 hours with Curdlan (25 μg/mL) or HKCA (106 particles/mL). The cells were lysed in passive lysis buffer, and luciferase activities were measured in the Dual-Luciferase Reporter Assay, according to the manufacturer’s instructions (Promega). For each experimental condition, the ratio of firefly RLU to *Renilla* RLU was calculated and plotted, along with the corresponding standard deviations.

### qPCR.

RNA was extracted with the Zymo Quick-RNA Microprep kit (R1051). Any genomic DNA was removed using Zymo Spin-Away filters (C10006-250-F). RNA was reversed-transcribed with the High-Capacity RNA-to-cDNA kit (4387406, Applied Biosystems) according to the manufacturer’s protocol. qPCR was performed on cDNA with TaqMan Fast Advanced Master Mix (4444557, Thermo Fisher Scientific) on a QuantStudio 7 Pro Real-Time PCR System (Applied Biosystems) with the following probes: CARD9 exons 2–3 (Hs01008677_g1) and GUSB (4326320E).

### Immunoblotting and IP.

Cells were washed with cold PBS and lysed in a buffer containing 50 mM Tris-HCl pH 7.4, 150 mM NaCl, 0.5% Triton X-100, and 2 mM EDTA supplemented with protease inhibitors (Complete Mini protease inhibitor cocktail, 4693124001, Roche) and phosphatase inhibitor cocktail (PhoStop, 4906837001, Roche). Lysates were incubated for 30 minutes at 4°C and mixed by vortex every 10 minutes. The cells were centrifuged for 20 minutes at 16,000*g* at 4°C, and the supernatant was collected for immunoblotting. Protein yield was determined with the Bradford protein assay (Bio-Rad), and equal amounts of total samples were separated by SDS-PAGE (4%–20% polyacrylamide gel). Proteins were transferred onto a PVDF membrane using a wet transfer system (Bio-Rad). The membrane was blocked by incubation with Intercept (PBS) Protein-Free Blocking buffer (927-90001, Licor) for 1 hour at room temperature. Membranes were probed with antibodies directed against C-term CARD9 (unconjugated, clone EPR6489, ab133560, Abcam), N-term CARD9 (unconjugated, clone A8, sc-374569, Santa Cruz Biotechnology), GAPDH (unconjugated, clone 6C5, sc-32233, Santa Cruz Biotechnology). Primary antibodies were detected by incubation with goat anti-rabbit IRDye 680RD (926-68071, Licor) and donkey anti-mouse IRDye 800CW (926-32212, Licor). Binding was detected with Odyssey CLx Imager (Licor). The Chameleon Duo Prestained Protein Ladder (928-60000, Licor) was used to provide a molecular weight marker. Images were analyzed with Image Studio software (Licor). For IP, cells were lysed in lysis buffer (0.5% Triton X-100, 20 mM HEPES pH 7.4, 150 mM NaCl, 12.5 mM b-glycerophosphate, 1.5 mM MgCl_2_) supplemented with protease inhibitors (Complete Mini protease inhibitor cocktail, 4693124001, Roche). Cell extracts were incubated overnight at 4°C with 1 μg of N-term CARD9 (unconjugated, clone A8, sc-374569, Santa Cruz Biotechnology) and 500 μL of Pierce Protein A/G Magnetic Beads (Thermo Fisher Scientific, C/N88802), prewashed, and resuspended in lysis buffer at a 1:1 ratio. Samples were separated by SDS-PAGE, and the proteins were transferred onto a PVDF membrane. The membrane was incubated with CARD9-HRP antibody (clone A8, sc-374569 HRP, Santa Cruz Biotechnology), and immunoreactive proteins were visualized by enhanced chemiluminescence.

### Deep immunophenotyping.

Cryopreserved PBMCs and their subpopulations were analyzed with a 28-color flow cytometry panel, as previously described (57). The following mAbs were used: anti-CD20 BUV805, anti-CD10 APC, anti-Vαβ TCR BUV737, anti-CD4 APCCy7, anti-CD25 PECy7, anti-CD27 PECy7, anti-CD27 PE, anti-CD45RA PerCpCy5, anti-CXCR5 BUV615, anti-IgG APC, anti-IgG BB660, anti-IgD BV480, anti-IgG BV605, anti-IgA1/A2 PECy5, anti-CD8 BUV496, anti-CD21 BUV563, anti-PD1 BV605, anti-IgM PerCPCy5.5, anti-IgM APC R700, anti-CD3 BV421, anti-IL-2 BV711, anti-IL-9 PerCPCy5.5, anti-IL-13 BV421, anti-IL-17F BV786, anti-IFN-γ BV605, anti-TNF BUV395, anti-CD19 BV711, anti-CD34 FITC, anti-CCR6 PE, anti-CD45RA BUV395, and anti-CXCR5 BV615 (all from Becton Dickinson); anti-CD20 Pacific blue, anti-CCR7 PECy7, anti-CD127 BV650, anti-IL-17A APCCy7, anti-CD20 BUV805, anti-CXCR3 BV421, and anti-CD3 BV570 (all from BioLegend); anti-CCR7 FITC (R&D Systems); anti-IL-4 PECy7, anti-IL-21 e660, and anti-IL-22 PE (all from Thermo Fisher Scientific).

### Isolation and functional characterization of human CD4^+^ T cells.

Naive and memory CD4^+^ T cells were isolated by excluding Tregs (CD25^hi^CD127^lo^) and then sorting CD45RA^+^CCR7^+^ cells and CD45RA^–^CCR7^+/–^ cells, ensuring greater than 98% purity of the recovered populations. Sorted naive and memory CD4^+^ T cells were cultured in 96-well round-bottom plates (40 × 10^3^ cells/well) under T_H_0 (T cell activation and expansion [TAE] beads [anti-CD2/CD3/CD28; Miltenyi Biotec] only), T_H_1 polarizing (TAE beads plus 50 ng/mL IL-12), T_H_2 polarizing (TAE beads plus 1 U/mL IL-4), T_H_9 polarizing (TAE beads plus 100 U/mL IL-4 and 2.5 ng/mL TGF-β), or T_H_17 polarizing (TAE beads plus 2.5 ng/mL TGF-β, 50 ng/mL IL-1b, 50 ng/mL IL-6, 50 ng/mL IL-21, 50 ng/mL IL-23) conditions. After 5 days, the supernatant was used for assessments of the secretion of IL-2, IL-4, IL-5, IL-6, IL-9, IL-10, IL-13, IL-17A, IL-17F, IFN-γ, and TNF-α with a cytometric bead array (BD Biosciences), and IL-22 by ELISA (PeproTech, 900-K246). Once the supernatant had been collected, the cells were stimulated with PMA (100 ng/mL, P8139-1 MG, Sigma Aldrich) with ionomycin (750 ng/mL, I0634-1MG, Sigma Aldrich) for 6 hours, and brefeldin A (10 mg/mL, B7651-5MG, Sigma Aldrich) was added after the first 2 hours of incubation. For the assessment of intracellular cytokine production, cells were incubated with conjugated mAbs directed against IFN-γ (BUV737, clone 4S.B3, 564620, BD Horizon), TNF-α (PerCP, clone Mab11, 502924, BioLegend), IL-9 (PE, clone MH9A3, 560807, BD Pharmingen), IL-13 (BV421, clone JES10-5A2, 563580, BD Horizon), IL-4 (AF488, clone 8D4-8, 500710, BioLegend), IL-17A (BV510, clone BL168, 512330, BioLegend), IL-17F (BV650, clone O33-782, 562264, BD Horizon), IL-2 (BV750, clone MQ1-17H12, 566361, BD Horizon), and IL-21 (eF660, clone eBio3A3-N2, 50-7219-42, Thermo Fisher Scientific), as previously described (58, 59). All cells were also stained with the Zombie UV fixable viability dye kit (423107, BioLegend). Cells and beads were acquired on a BD FACSymphony A5 Cell Analyzer (BD Biosciences) and analyzed with FlowJo Software or FCAP Array software (CBA).

### Activation-induced cell marker assay to detect Ag-specific CD4^+^ T cells.

We adapted the assay first described by Zaunders and colleagues (49). Isolated cryopreserved PBMCs were cultured in vitro for 48 hours in 96-well plates (10^6^ cells/mL) in media only or with HKCA (tlrl-hkca, InvivoGen; 10^6^ particles/mL) or PHA (lectin from *Phaseolus vulgaris*, Sigma-Aldrich L1668; 5 μg/mL) at 37°C, under an atmosphere containing 5% CO_2_. Brefeldin A (B7651-5MG, Sigma Aldrich, 10 mg/mL) was added to the culture for the last 4 hours of incubation. Harvested cells were incubated with conjugated mAbs directed against CD4 (BUV737, clone SK3, 612748, BD Horizon), CD8 (BUV395, clone RPA-T8, 563795, BD Horizon), CD25 (FITC, clone 2A3, 347643, BD Biosciences), CD45RA (PerCPCy5.5, clone HI100, 45-0458-42, eBioscience), CCR7/CD197(PECy7, clone G043H7, 353226, BioLegend), CD137 (APC, clone REA765, 130-110-764, Miltenyi Biotec), and OX40 (PE, clone ACT35, 12-1347-42, eBioscience). For the assessment of intracellular cytokine production, cells were incubated with conjugated mAbs directed against IFN-γ (BUV737, clone 4S.B3, 564620, BD Horizon), TNF-α (PerCP, clone Mab11, 502924, BioLegend), IL-17A (BV510, clone BL168, 512330, BioLegend), IL-17F (BV650, clone O33-782, 562264, BD Horizon), and IL-2 (BV750, clone MQ1-17H12, 566361, BD Horizon). All cells were also stained with the Zombie UV fixable viability dye kit (423107, BioLegend). Cells were acquired on a BD FACSymphony A5 Cell Analyzer (BD Biosciences) and analyzed with FlowJo.

### Fibroblast activation.

Primary fibroblasts were plated in 48-well plates at a density of 50,000 cells/well in 0.5 mL DMEM/10% FBS per well. The cells were left unstimulated or were stimulated for 24 hours with TNF-α (50 ng/mL), LPS (10 ng/mL), Curdlan (100 μg/mL), zymosan depleted (10 μg/mL), HKCA (10^6^ particles/mL), and polyinosinic-polycytidylic acid (25 μg/mL). Supernatants were collected after 24 hours and stored at –20°C until their use for cytokine (IL-6 and TNF-α) determinations by cytometric bead array (BD Biosciences).

### Statistics.

Significant differences were determined using GraphPad Prism 8. Data were tested for normal distribution of variables and are displayed as mean ± SD unless otherwise noted. Measurements between 2 groups were performed with a 2-tailed Student’s *t* test if normally distributed, or Mann-Whitney *U* test otherwise. Groups of 3 or more were analyzed by 1-way ANOVA or Kruskal-Wallis test or paired 1-way ANOVA or Friedman test. Grouped analyses were interrogated by 2-way ANOVA with post hoc multiple-comparison tests. A *P* value less than 0.05 was considered significant.

### Study approval.

Buffy coats from healthy donors were purchased from the Australian Red Cross Blood Service. Peripheral blood was collected from patients with CARD9 deficiency and their relatives. This study was approved by the Sydney Local Health District RPAH Zone Human Research Ethics Committee and Research Governance Office, Royal Prince Alfred Hospital, Camperdown, New South Wales, Australia (protocol X16-0210/LNR/16/RPAH/257); St Vincent’s Hospital Human Research Ethics Committee, Darlinghurst, New South Wales, Australia (2021/ETH000365); and the South Western Sydney Human Research Ethics Committee, Liverpool Hospital, Liverpool, New South Wales, Australia (2019/ETH13400). Written informed consent was obtained from participants. Experiments using samples from patients were conducted in accordance with local regulations and with the approval of the IRBs of corresponding institutions. Patients signed informed consent regarding publishing their data.

### Data availability.

The Supporting Data Values file is available upon request to the corresponding authors.

## Author contributions

EDM, SGT, and AK conceived the study. EDM and SGT designed the study; EDM conducted experiments. CGEH, TAW, CWTC, FH, EKD, JLC, AP, and AK provided patient material and clinical information. JLC and AP provided methodologies, reagents, and fibroblast cell lines. JJL and VL provided the histopathological data. CSM developed, optimized, and implemented protocols for assessing human PBMC phenotypes and CD4^+^ T cell responses in vitro. CSM and SGT supervised the laboratory study. SGT funded the project. EDM and SGT wrote the initial draft of the original and revised manuscripts; all authors contributed to the final version of the manuscript and approved its submission.

## Conflict of interest

The authors have declared that no conflict of interest exists.

## Funding support

This work is the result of NIH funding, in whole or in part, and is subject to the NIH Public Access Policy. Through acceptance of this federal funding, the NIH has been given a right to make the work publicly available in PubMed Central.

- Early-Mid Career Research Fellowship from the Department of Health of the New South Wales Government of Australia to CSM.
- National Health and Medical Research Council (NHMRC) of Australia Investigator grant (level 1) (2017463) to CSM.
- NHMRC Principal Research Fellowship (1042925) to SGT.
- NHMRC Program grant (1113904) to SGT.
- NHMRC Investigator grants (Leadership 3; 1176665 and 2034593) to SGT.
- NHMRC Investigator grant (Leadership 2; 2026131) to EKD.
- Jeffrey Modell Foundation to the CIRCA investigators (EDM, EKD, CSM, SGT, AK).
- CORIO Foundation to the CIRCA investigators (EDM, EKD, CSM, SGT, AK).
- John Brown Cook Foundation to the CIRCA investigators (EDM, EKD, CSM, SGT, AK).
- St Vincent’s Clinic Foundation grant to AK.
- Howard Hughes Medical Institute to Laboratory of Human Genetics of Infectious Diseases.
- The Rockefeller University to Laboratory of Human Genetics of Infectious Diseases.
- St. Giles Foundation to Laboratory of Human Genetics of Infectious Diseases.
- NIH (R01AI127564) to Laboratory of Human Genetics of Infectious Diseases.
- National Center for Advancing Translational Sciences to Laboratory of Human Genetics of Infectious Diseases.
- NIH Clinical and Translational Science Award program (UL1TR001866) to Laboratory of Human Genetics of Infectious Diseases.
- French Agence Nationale de la Recherche (ANR) under France 2030 program (ANR-10-IAHU-01) to Laboratory of Human Genetics of Infectious Diseases.
- Integrative Biology of Emerging Infectious Diseases Laboratory of Excellence (ANR-10-LABX-62-IBEID) to Laboratory of Human Genetics of Infectious Diseases.
- ANR LTh-MSMD-CMCD (ANR-18-CE93-0008) project to Laboratory of Human Genetics of Infectious Diseases.
- French Foundation for Medical Research (FRM) (EQU202503020018) to Laboratory of Human Genetics of Infectious Diseases.
- Square Foundation to Laboratory of Human Genetics of Infectious Diseases.
- Grandir-Fonds de solidarité pour l’enfance to Laboratory of Human Genetics of Infectious Diseases.
- William E. Ford (General Atlantic’s chairman and chief executive officer) to Laboratory of Human Genetics of Infectious Diseases.
- Gabriel Caillaux (General Atlantic’s copresident, managing director, and head of business at Europe, Middle East, and Africa) to Laboratory of Human Genetics of Infectious Diseases.
- General Atlantic Foundation, Institut National de la Santé et de la Recherche Médicale (INSERM) to Laboratory of Human Genetics of Infectious Diseases.
- Paris Cité University to Laboratory of Human Genetics of Infectious Diseases.

## Supplementary Material

Supplemental data

Unedited blot and gel images

Supporting data values

## Figures and Tables

**Table 1:**
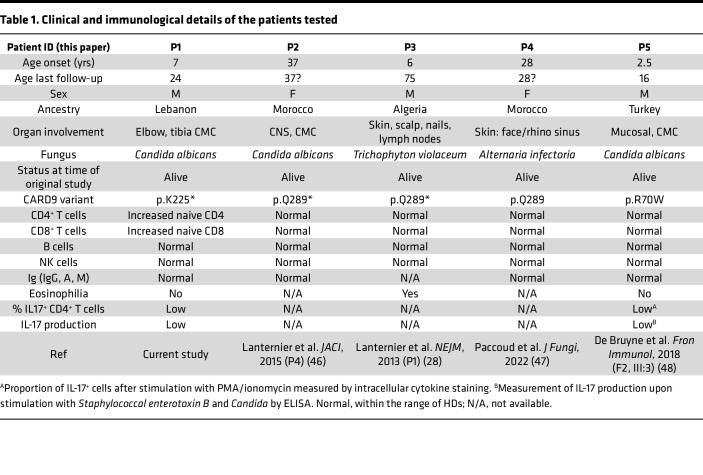
Clinical and immunological details of the patients tested

## Version 1 04/22/2026

Electronic publication
