# Supplementary material for: Inherited human CARD9 deficiency impairs lymphoid cell, but not fibroblast, IL-17–mediated immunity
Source: JCI Insight. 2026 Apr 22;11(8):e190875. doi: 10.1172/jci.insight.190875 (PMC13135412; doi:10.1172/jci.insight.190875)

Supplemental Materials

Inherited human CARD9 deficiency impairs lymphoid cell, but not fibroblast, IL-17 mediated immunity

Erika Della Mina[1,2,3], Carlos El Haddad[4,5,6,], Timothy West[7], Clara W.T. Chung[8,9], Jing Jing Li[10], Vivienne Lea[10], Elissa K Deenick[1,2,3], Filomeen Haerynck[11,12], Jean-Laurent Casanova[13,14,15,16,17], Anne Puel[13,14,15], Cindy S Ma[1,2,3], Stuart G Tangye[1,2,3,#], Alisa Kane[3,6,7,18,19,#]

Table of contents:

Supp Table 1. (page 2)

Supplemental Fig1 (page 3)

Supplemental Fig2 (page 4)

Supplemental Fig3 (page 5)

Supp Table 1. List of 278 unique genes tested for P1

Genes							
ACP5	ACTB	ADA	ADA2	ADAR	AICDA	AIRE	AK2
AP3B1	AP3D1	APOL1	ARPC1B	ATM	ATP6AP1	B2M	BACH2
BCL10	BCL11B	BLM	BLNK	BLOC1S6	BTK	CARD11	CARD8
CARMIL2	CASP10	CASP8	CCBE1	CD19	CD27	CD3D	CD3E
CD3G	CD40	CD40LG	CD70	CD79A	CD79B	CD81	CD8A
CDCA7	CEBPE	CFTR	CHD7	CIITA	CLCN7	CLPB	CORO1A
CR2	CSF2RA	CSF2RB	CSF3R	CTC1	CTLA4	CTPS1	CTSC
CXCR4	CYBA	CYBB	DCLRE1B	DCLRE1C	DKC1	DNAJC21	DNMT3B
DOCK2	DOCK8	ELANE	EPGS	ERCC6L2	EXTL3	FAAP24	FADD
FAS	FASLG	FAT4	FCGR3A	FERMT3	FOXN1	FOXP3	FPR1
G6PC3	G6PD	GATA2	GFI1	GINS1	HAX1	HELLS	HMOX1
HMOX2	HYOU1	ICOS	IFIH1	IFNAR2	IFNGR1	IFNGR2	IGHM
IGKC	IGLL1	IKBKB	IKBKG	IKZF1	IL10	IL10RA	IL10RB
IL12B	IL12RB1	IL17F	IL17RA	IL17RC	IL21R	IL2RA	IL2RG
IL7R	INO80	IRAK1	IRAK4	IRF2BP2	IRF3	IRF7	IRF8
ISG15	ITCH	ITGB2	ITK	JAGN1	JAK1	JAK3	KDM6A
KMT2D	KRAS	LAMTOR2	LCK	LIG1	LIG4	LRBA	LYST
MAGT1	MALT1	MAP3K14	MCM4	MKL1	MOGS	MS4A1	MSH6
MSN	MTHFD1	MYD88	MYSM1	NBAS	NBN	NCF1	NCF2
NCF4	NCSTN	NFAT5	NFKB1	NFKB2	NFKBIA	NHP2	NOP10
NRAS	NSMCE3	ORAI1	OSTM1	PARN	PEPD	PGM3	PIK3CD
PIK3R1	PLEKHM1	PMS2	PNP	POLE	POLE2	PRF1	PRKCD
PRKDC	PSEN1	PSENEN	PTEN	PTPRC	RAC2	RAG1	RAG2
RANBP2	RASGRP1	RBCK1	RELB	RFX5	RFXANK	RFXAP	RHOH
RMRP	RNASEH2A	RNASEH2B	RNASEH2C	RNF168	RNF31	RNU4ATAC	RORC
RPSA	RTEL1	SAMD9	SAMD9L	SAMHD1	SBDS	SEMA3E	SH2D1A
SLC35C1	SLC37A4	SLC46A1	SLC7A7	SMARCAL1	SMARCD2	SNX10	SP110
SPINK5	STAT1	STAT2	STAT3	STAT5B	STIM1	STK4	STN1
STX11	STXBP2	TAP1	TAP2	TAPBP	TAZ	TBK1	TBX1
TCF3	TCN2	TERC	TERT	TFRC	TINF2	TIRAP	TMC6
TMC8	TMEM173	TNFRSF11A	TNFRSF13B	TNFRSF13C	TNFRSF4	TNFSF11	TNFSF12
TPP1	TPP2	TRAC	TRAF3	TRAF3IP2	TREX1	TRNT1	TTC37
TTC7A	TYK2	UNC119	UNC13D	UNC93B1	UNG	USB1	VPS13B
VPS45	WAS	WDR1	WIPF1	WRAP53	XIAP	ZAP70	ZBTB24

Sup Fig. 1 Whole blood stimulation with Dectin-1 ligands

(**A**) *CARD9* gDNA sequencing chromatograms, for a healthy donor (HD, top panel), P1 (middle panel) and P1's mother (bottom panel) for the exon 5 region corresponding to P1 missense variant c.673A>T. (**B**) HEK293T cells were untransfected (NT) or transfected with plasmids encoding WT, K225*/K225*, or Q289*/Q289* CARD9 or the empty vector (EV). Cells were stained with Abs against CARD9 N-term. The histogram in panel (**A)** depicts the frequency of alive CARD9-expressing cells in each condition tested. (**C**) IL-6 (left) and TNF-α (right) production by whole blood after 24 hours of stimulation with LPS (TLR-4 agonist), heat-killed *S aureus* (HKSA, TLR-2 agonist), Zymosan Depleted, Curdlan, heat-killed *C albicans* (HKCA) (all Dectin-1 agonists), and PMA plus ionomycin for healthy donors (n=6, white bars) and P1(grey bars). Each data point corresponds to individual healthy donors or CARD9-deficient patient samples. The mean ± SD for 2 independent experiments is shown.

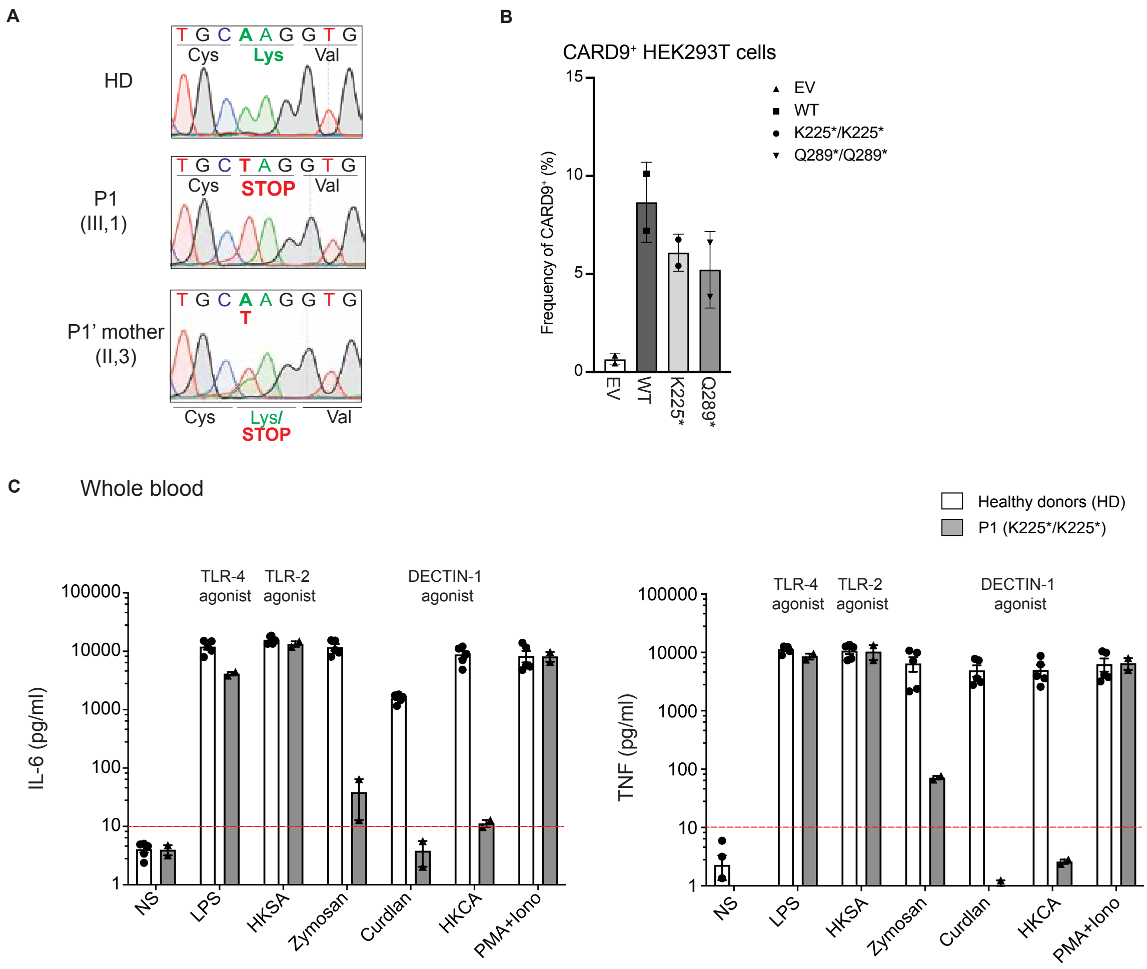

Sup Fig. 2 B cells and NK cells phenotype

PBMCs from healthy donors (HD, n=15, white bars), P1 (homozygous CARD9 K225* variant; light grey bars), previous reported P2 and P3 (CARD9 Q289*/Q289*; dark grey bars) and the heterozygous P1's mother (CARD9 K225*/WT, red circles among HDs) were stained to determine the proportions of (**A**) transitional (CD27$^-$CD10$^+$), naïve (CD27$^-$CD10$^-$) and memory (CD27$^+$CD10$^-$) B cells; (**B)** CD21 high (CD20$^+$CD21^{high}) and CD21low (CD20$^+$CD21^{low}) B cells; (**C**) Ig switched memory B cells (IgD$^-$IgM$^-$CD27$^+$); (**D**) NK cells subsets (CD56^{high}/CD56^{low}). Each data point corresponds to individual healthy donors or CARD9-deficient patients. The mean ± SD for 3 independent experiments is shown.

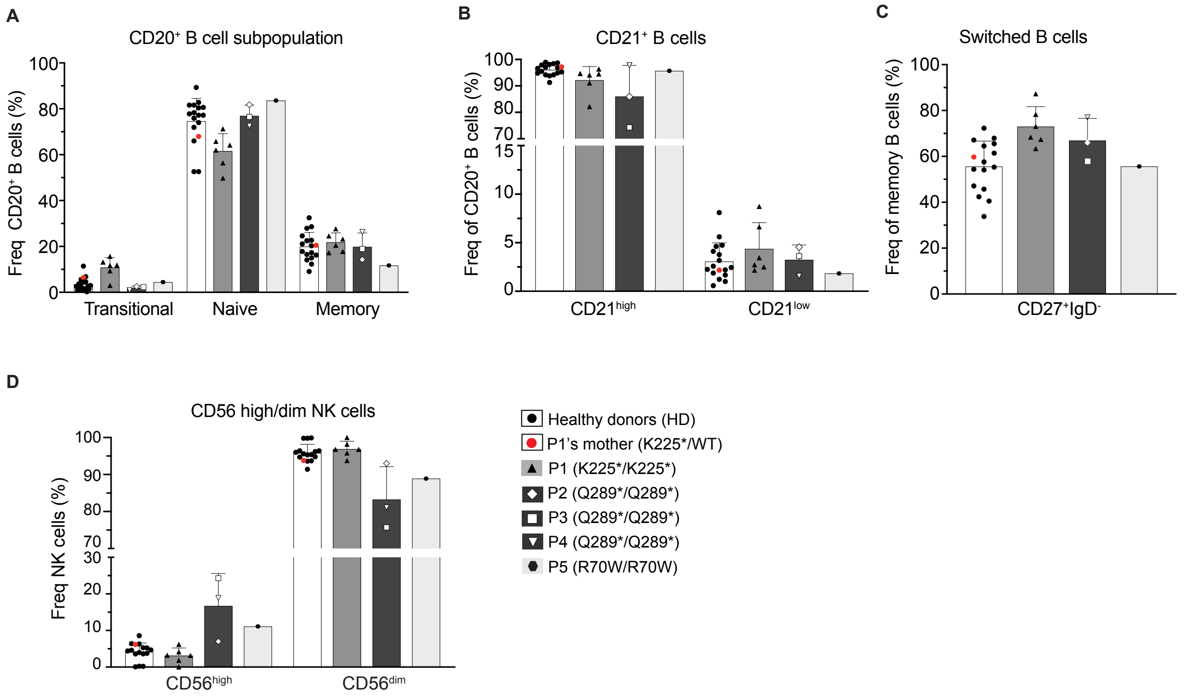

Sup Fig. 3 Cytokine intracellular expression by memory and naïve $CD4^+$ T cells

Sort-purified (A-F) memory and (G-I) naïve $CD4^+$ T cells from healthy donors (HD, n = 10) and P1 (n = 3) were cultured for 5 d with TAE beads under T_H0, T_H1, T_H2, T_H9, T_H17-polarizing conditions. Cells were then restimulated with PMA/ionomycin for 6 hours in the presence of Brefeldin A for the last 4 hours. After this time, intracellular expression of (**A,F**) IFNγ and TNFα (**B, G**) IL-4, IL-13, and IL-5, (**C,H**) IL-9 (**D**) IL-17A, and IL-17F, were determined by intracellular staining. Each point represents a different individual. The mean ± SEM for 3 independent experiments is shown.

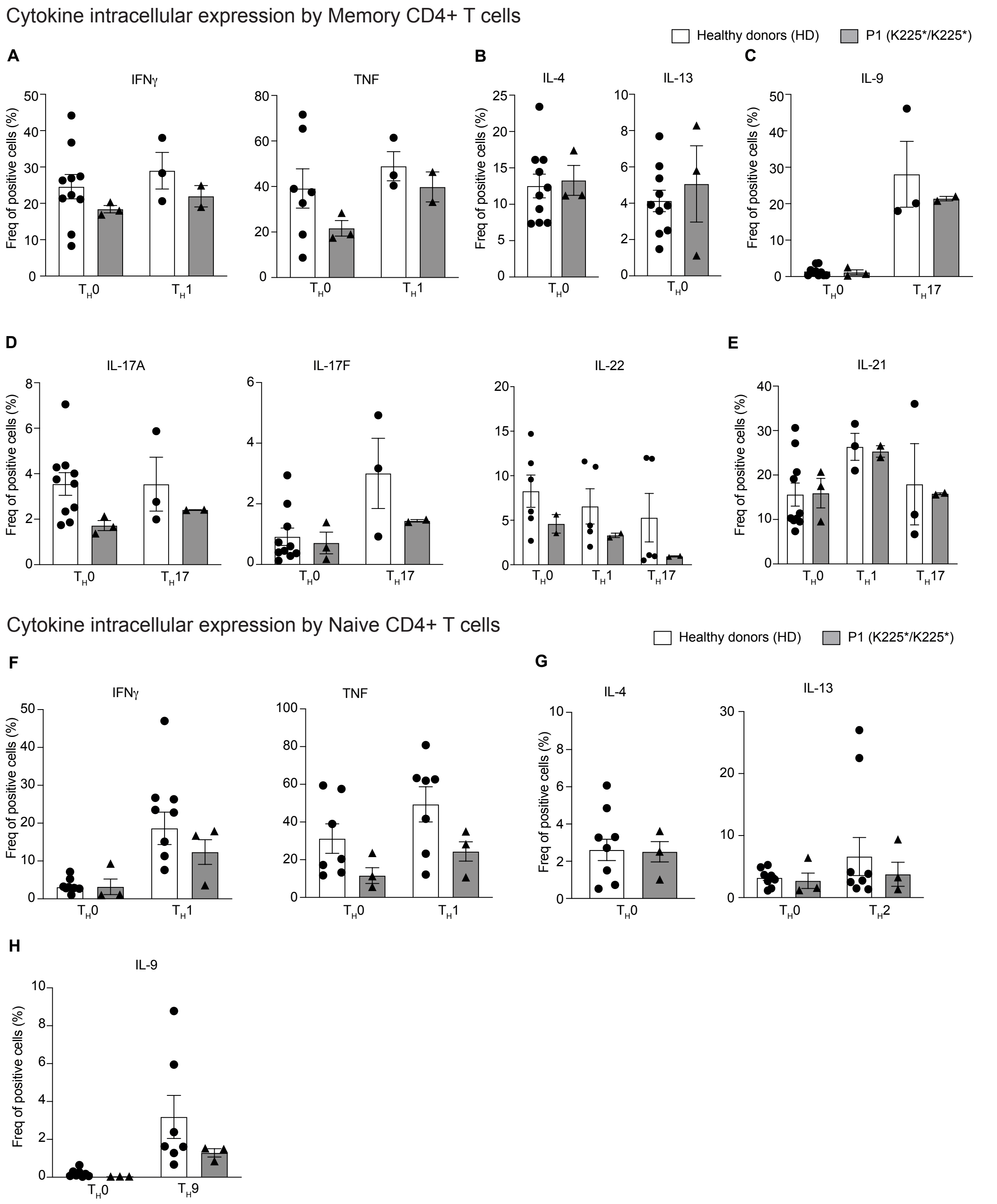

Supplement: Supplemental data [file jciinsight-11-190875-s083.pdf]
