# Supplementary material for: Inherited human CARD9 deficiency impairs lymphoid cell, but not fibroblast, IL-17–mediated immunity
Source: JCI Insight. 2026 Apr 22;11(8):e190875. doi: 10.1172/jci.insight.190875 (PMC13135412; doi:10.1172/jci.insight.190875)
Supplement: Unedited blot and gel images [file jciinsight-11-190875-s084.pdf]

Full unedited gel for Figure 2A

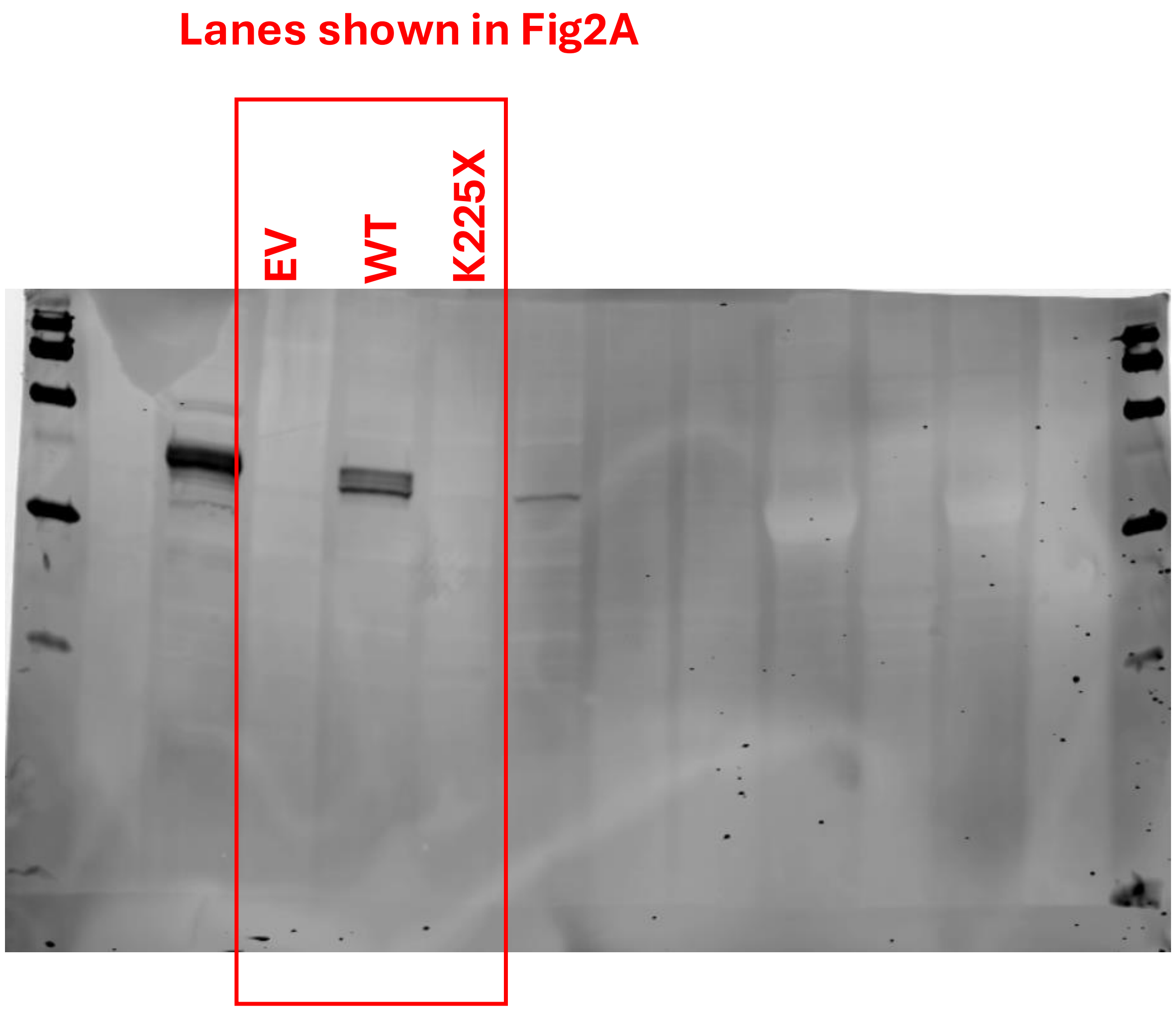

Anti-CARD9 C-term

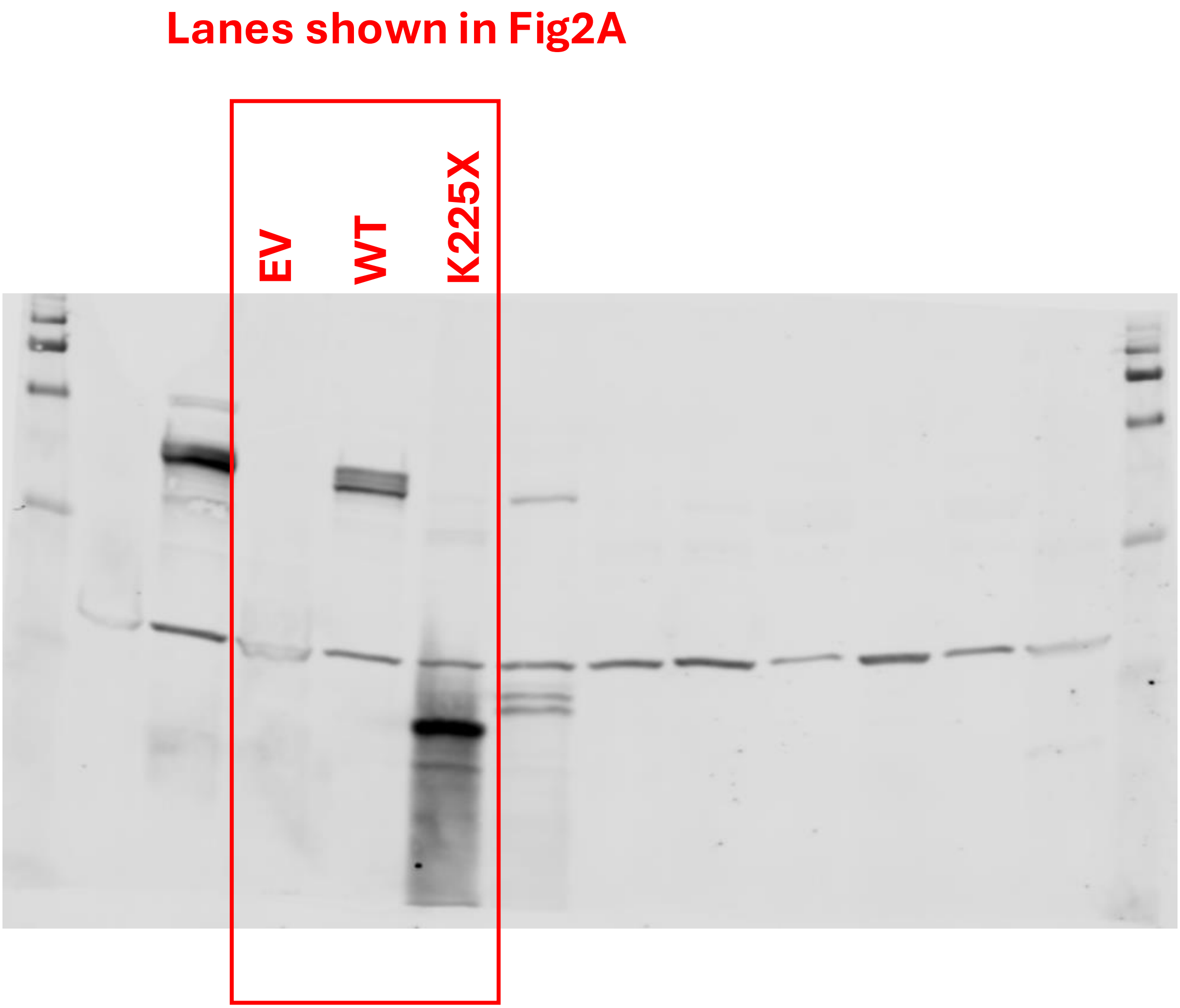

Anti-CARD9 N-term
anti-GAPDH

Full unedited gel Figure 2F

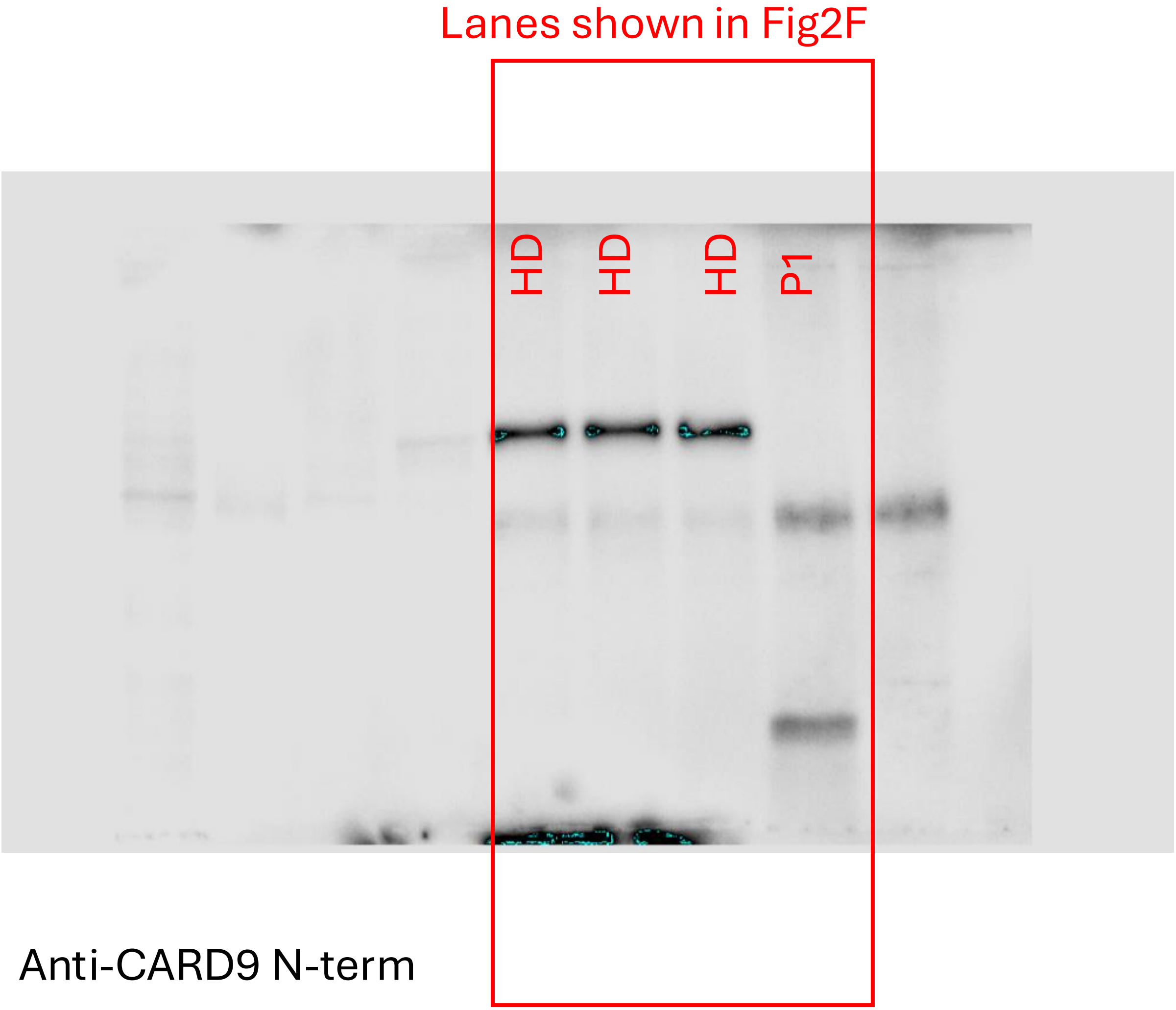

Anti-CARD9 N-term

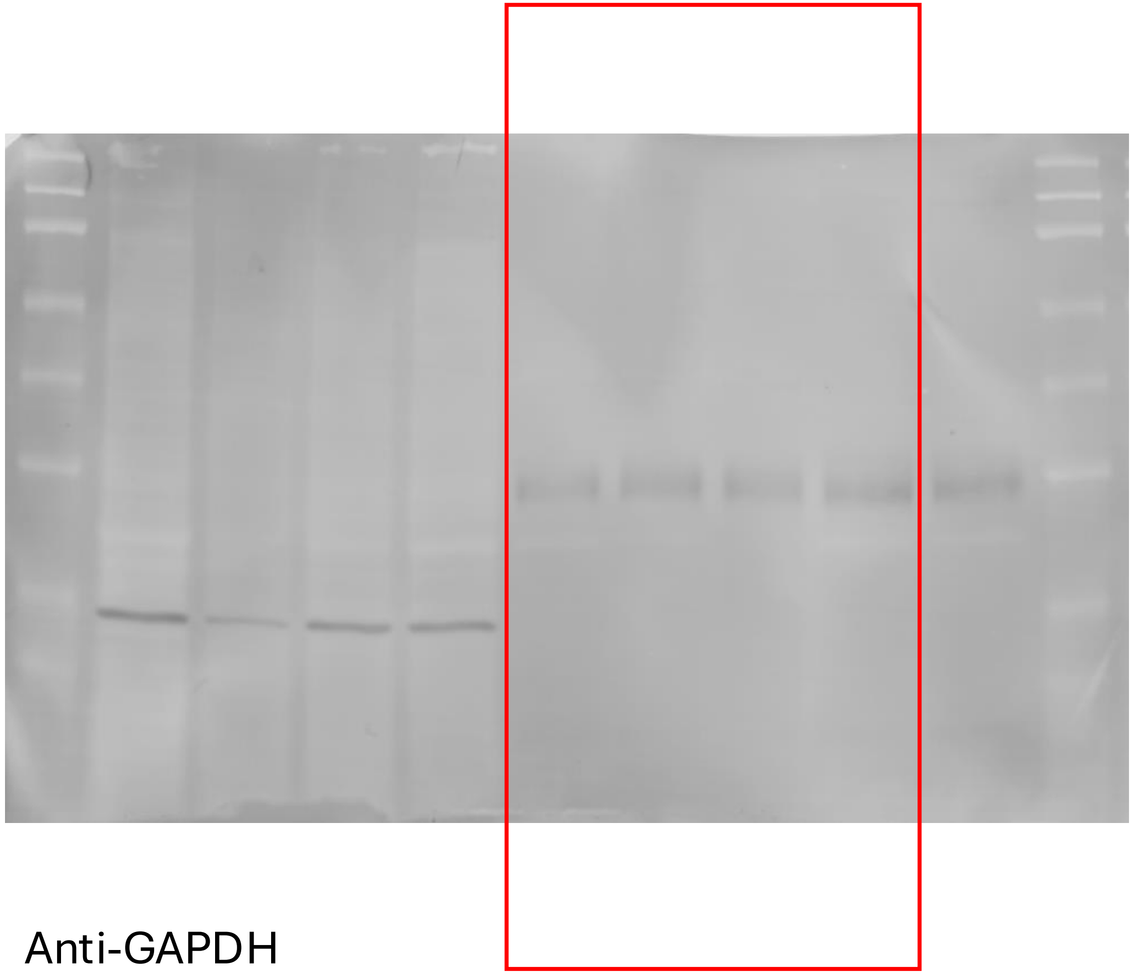

Anti-GAPDH
